# Diagnosis of obstructive coronary artery disease using computed tomography angiography in patients with stable chest pain depending on clinical probability and in clinically important subgroups: meta-analysis of individual patient data

**DOI:** 10.1136/bmj.l1945

**Published:** 2019-06-12

**Authors:** Robert Haase, Peter Schlattmann, Pascal Gueret, Daniele Andreini, Gianluca Pontone, Hatem Alkadhi, Jörg Hausleiter, Mario J Garcia, Sebastian Leschka, Willem B Meijboom, Elke Zimmermann, Bernhard Gerber, U Joseph Schoepf, Abbas A Shabestari, Bjarne L Nørgaard, Matthijs F L Meijs, Akira Sato, Kristian A Ovrehus, Axel C P Diederichsen, Shona M M Jenkins, Juhani Knuuti, Ashraf Hamdan, Bjørn A Halvorsen, Vladimir Mendoza-Rodriguez, Carlos E Rochitte, Johannes Rixe, Yung Liang Wan, Christoph Langer, Nuno Bettencourt, Eugenio Martuscelli, Said Ghostine, Ronny R Buechel, Konstantin Nikolaou, Hans Mickley, Lin Yang, Zhaqoi Zhang, Marcus Y Chen, David A Halon, Matthias Rief, Kai Sun, Beatrice Hirt-Moch, Hiroyuki Niinuma, Roy P Marcus, Simone Muraglia, Réda Jakamy, Benjamin J Chow, Philipp A Kaufmann, Jean-Claude Tardif, Cesar Nomura, Klaus F Kofoed, Jean-Pierre Laissy, Armin Arbab-Zadeh, Kakuya Kitagawa, Roger Laham, Masahiro Jinzaki, John Hoe, Frank J Rybicki, Arthur Scholte, Narinder Paul, Swee Y Tan, Kunihiro Yoshioka, Robert Röhle, Georg M Schuetz, Sabine Schueler, Maria H Coenen, Viktoria Wieske, Stephan Achenbach, Matthew J Budoff, Michael Laule, David E Newby, Marc Dewey

**Affiliations:** 1 Department of Radiology, Charité - Universitätsmedizin Berlin, Charitéplatz 1, 10117 Berlin, Germany; 2 Institute of Medical Statistics, Computer Sciences and Data Science, University Hospital of Friedrich Schiller University Jena, Jena, Germany; 3 Department of Cardiology, Henri Mondor Hospital, University Paris Est Créteil, Créteil, France; 4 Department of Cardiology and Radiology, Centro Cardiologico Monzino IRCCS, University of Milan, Milan, Italy; 5 Centro Cardiologico Monzino, IRCCS, Milan, Italy; 6 Department of Radiology, University Hospital Zurich, Zurich, Switzerland; 7 Medizinische Klinik und Poliklinik I, Ludwig-Maximilians-Universität München, Munich, Germany; 8 Department of Cardiology, Montefiore, University Hospital for the Albert Einstein College of Medicine, NY, USA; 9 Department of Radiology, Kantonsspital St Gallen, St Gallen, Switzerland; 10 Department of Cardiology, Erasmus University Medical Centre, Rotterdam, Netherlands; 11 Department of Cardiology, Clinique Universitaire St Luc, Institut de Recherche Clinique et Expérimentale, Brussels, Belgium; 12 Department of Radiology and Radiological Science, Medical University of South Carolina, Charleston, SC, USA; 13 Modarres Hospital, Shahid Beheshti University of Medical Sciences, Tehran, Iran; 14 Department of Cardiology, Aarhus Universtity Hostipal, Aarhus, Denmark; 15 Department of Cardiology, University Medical Centre Utrecht, Utrecht, Netherlands; 16 Cardiovascular Division, Faculty of Medicine, University of Tsukuba, Tsukuba, Japan; 17 Department of Cardiology, Odense University Hospital, Odense, Denmark; 18 Department of Cardiology, Glasgow Royal Infirmary and Stobhill Hospital, Glasgow, UK; 19 Turku University Hospital and University of Turku, Turku, Finland; 20 Department of Cardiovascular Imaging, Department of Cardiology, Rabin Medical Center, Sackler Faculty of Medicine, Tel-Aviv University, Tel-Aviv, Israel; 21 Medical Department, Ostfold Hospital Trust, Grålum, Norway; 22 Department of Cardiology, National Institute of Cardiology and Cardiovascular Surgery, Havana, Cuba; 23 Heart Institute, InCor, University of São Paulo Medical School, São Paulo, Brazil; 24 Department of Cardiology, Kerckhoff Heart Centre, Bad Nauheim, Germany; 25 Medical Imaging and Radiological Sciences, College of Medicine, Chang Gung University, Chang Gung Memorial Hospital at Linkou, Taoyaun City, Taiwan; 26 Heart and Diabetes Center NRW in Bad Oeynhausen, University Clinic of the Ruhr-University Bochum, Bochum, Germany; 27 Department of Cardiology, Centro Hospitalar de Vila Nova de Gaia, Vila Nova de Gaia, Portugal; 28 Department of Internal Medicine, University of Rome Tor Vergata, Rome, Italy; 29 Department of Cardiology, Centre Chirurgical Marie Lannelongue, Le Plessis Robinson, France; 30 Department of Nuclear Medicine, University Hospital Zurich, Zurich, Switzerland; 31 Department of Diagnostic and Interventional Radiology, University Hospital of Tübingen, Tübingen, Germany; 32 Department of Cardiology, Odense University Hospital, Odense, Denmark; 33 Department of Radiology, Beijing Anzhen Hospital, Beijing, China; 34 National Heart and Blood Institute, National Institutes of Health, Bethesda, MD, USA; 35 Cardiovascular Clinical Research Unit, Lady Davis Carmel Medical Center, Haifa, Israel; 36 Department of Radiology, Baotou Central Hospital, Inner Mongolia Province, China; 37 St Luke’s International Hospital, Tokyo, Japan; 38 Department of Cardiology, S Chiara Hospital, Trento, Italy; 39 Department of Cardiology, University Hospital Pitié-Salpêtrière, Paris, France; 40 University of Ottawa, Heart Institute, Ottawa, Ontario, Canada; 41 Montreal Heart Institute, Université de Montréal, Montréal, Canada; 42 Albert Einstein Hospital, São Paulo, Brazil; 43 The Heart Centre, Rigshospitalet, University of Copenhagen, Copenhagen, Denmark; 44 Department of Diagnostic Imaging and Interventional Radiology, Bichat University Hospital, Paris, France; 45 Division of Cardiology, Johns Hopkins Hospital, Johns Hopkins University, Baltimore, MD, USA; 46 Mie University Hospital, Tsu, Japan; 47 BIDMC/Harvard Medical School, Department of Cardiology, Beth Israel Deaconess Medical Center, Harvard University, Boston, MA, USA; 48 Department of Radiology, Keio University Hospital, Tokyo, Japan; 49 Department of Radiology, Mount Elizabeth Hospital, Singapore; 50 Department of Radiology, University of Ottawa, Ottawa, Ontario, Canada; 51 Department of Cardiology, Leiden University Medical Centre, Leiden, Netherlands; 52 Department of Medical Imaging, Western University, London, Ontario, Canada; 53 National Heart Centre, Singapore, Singapore; 54 Iwate Medical University, Morioka, Japan; 55 Department of Cardiology, Friedrich-Alexander University Erlangen-Nuremberg, Erlangen, Germany; 56 University of California Los Angeles, Los Angeles, CA, USA; 57 British Heart Foundation, University of Edinburgh, Edinburgh, UK

## Abstract

**Objective:**

To determine whether coronary computed tomography angiography (CTA) should be performed in patients with any clinical probability of coronary artery disease (CAD), and whether the diagnostic performance differs between subgroups of patients.

**Design:**

Prospectively designed meta-analysis of individual patient data from prospective diagnostic accuracy studies.

**Data sources:**

Medline, Embase, and Web of Science for published studies. Unpublished studies were identified via direct contact with participating investigators.

**Eligibility criteria for selecting studies:**

Prospective diagnostic accuracy studies that compared coronary CTA with coronary angiography as the reference standard, using at least a 50% diameter reduction as a cutoff value for obstructive CAD. All patients needed to have a clinical indication for coronary angiography due to suspected CAD, and both tests had to be performed in all patients. Results had to be provided using 2×2 or 3×2 cross tabulations for the comparison of CTA with coronary angiography. Primary outcomes were the positive and negative predictive values of CTA as a function of clinical pretest probability of obstructive CAD, analysed by a generalised linear mixed model; calculations were performed including and excluding non-diagnostic CTA results. The no-treat/treat threshold model was used to determine the range of appropriate pretest probabilities for CTA. The threshold model was based on obtained post-test probabilities of less than 15% in case of negative CTA and above 50% in case of positive CTA. Sex, angina pectoris type, age, and number of computed tomography detector rows were used as clinical variables to analyse the diagnostic performance in relevant subgroups.

**Results:**

Individual patient data from 5332 patients from 65 prospective diagnostic accuracy studies were retrieved. For a pretest probability range of 7-67%, the treat threshold of more than 50% and the no-treat threshold of less than 15% post-test probability were obtained using CTA. At a pretest probability of 7%, the positive predictive value of CTA was 50.9% (95% confidence interval 43.3% to 57.7%) and the negative predictive value of CTA was 97.8% (96.4% to 98.7%); corresponding values at a pretest probability of 67% were 82.7% (78.3% to 86.2%) and 85.0% (80.2% to 88.9%), respectively. The overall sensitivity of CTA was 95.2% (92.6% to 96.9%) and the specificity was 79.2% (74.9% to 82.9%). CTA using more than 64 detector rows was associated with a higher empirical sensitivity than CTA using up to 64 rows (93.4% *v* 86.5%, P=0.002) and specificity (84.4% *v* 72.6%, P<0.001). The area under the receiver-operating-characteristic curve for CTA was 0.897 (0.889 to 0.906), and the diagnostic performance of CTA was slightly lower in women than in with men (area under the curve 0.874 (0.858 to 0.890) *v* 0.907 (0.897 to 0.916), P<0.001). The diagnostic performance of CTA was slightly lower in patients older than 75 (0.864 (0.834 to 0.894), P=0.018 *v* all other age groups) and was not significantly influenced by angina pectoris type (typical angina 0.895 (0.873 to 0.917), atypical angina 0.898 (0.884 to 0.913), non-anginal chest pain 0.884 (0.870 to 0.899), other chest discomfort 0.915 (0.897 to 0.934)).

**Conclusions:**

In a no-treat/treat threshold model, the diagnosis of obstructive CAD using coronary CTA in patients with stable chest pain was most accurate when the clinical pretest probability was between 7% and 67%. Performance of CTA was not influenced by the angina pectoris type and was slightly higher in men and lower in older patients.

**Systematic review registration:**

PROSPERO CRD42012002780.

## Introduction

It is currently unclear in which subgroups of patients with suspected coronary artery disease (CAD) computed tomography angiography (CTA) has highest diagnostic clinical performance. Current guidelines recommend choosing the type of first line imaging test by taking the pretest probability of CAD into account, because it substantially affects diagnostic accuracy.^1^ ^2^ ^3^ According to the most recent recommendation of the National Institute for Health and Care Excellence,^4^ coronary CTA should be the primary imaging test in patients with suspected CAD and possible angina, while the guidelines of the European Society of Cardiology on the management of CAD recommend considering CTA only in patients with a CAD pretest probability of 15-50%.^3^ ^4^ Moreover, little is known about CTA’s diagnostic performance in clinically important patient subgroups such as sex, age, and angina pectoris type and its association with the estimated clinical pretest probability of CAD.

Optimising the use of diagnostic imaging tests in patients with suspected CAD is crucial, given that about two thirds of invasive coronary angiograms performed in Europe and the United States show no evidence of obstructive CAD and increasing use of cardiac imaging tests poses a burden on healthcare costs.^5^ ^6^ CTA has the potential to reliably exclude obstructive CAD,^7^ ^8^ while halving the events of coronary heart disease after five years of follow-up^9^ and improving the diagnostic yield of coronary angiography.^8^ ^10^ Its implementation as a first line diagnostic imaging test in patients with suspected CAD remains controversial. Since available diagnostic accuracy studies of CTA are moderate in size, data pooling can provide a more accurate assessment of its diagnostic performance. Individual patient data allow researchers to evaluate clinically important subgroups and individually estimate the pretest probability and to determine its effect on the diagnostic test performance of CTA. With the rationale to define the clinical context and clinical probability in which CTA has highest discriminative ability to diagnose or rule out CAD, we formed the COME-CCT (Collaborative Meta-Analysis of Cardiac CT) Consortium to pool patient level data from diagnostic accuracy studies of CTA enrolling patients with a clinical indication for coronary angiography as the reference standard for angiographic CAD.^11^ This work will help clinicians identify those patients with stable chest pain for whom CTA is most suitable.

## Methods

### Study design and main objectives

COME-CCT is a collaborative meta-analysis using individual patient data (IPD) to summarise the published and unpublished evidence on the diagnostic performance of cardiac CTA, and the protocol has been published.^11^ The main objective was to assess the influence of the clinical pretest probability of CAD on the diagnostic accuracy of cardiac CTA in order to define CTA’s clinical discriminative ability for diagnosing or ruling out CAD depending on clinical risk. Therefore, we used a no-treat/treat threshold approach to define the pretest probability range for which CTA has highest diagnostic value and, vice versa, for which CTA is not appropriate, to better decide which patients to offer the test to.^12^ Positive and negative predictive values (PPVs and NPVs) were chosen as primary outcome measures as a function of pretest probability.^13^ Finally, the influence of sex, age, angina pectoris type, and number of CT detector rows on the diagnostic performance of coronary CTA was analysed in this primary outcomes publication. We used the PRISMA (preferred reporting items for systematic reviews and meta-analyses) statement for IPD systematic reviews for reporting this collaborative meta-analysis (checklist in web appendix 1).^14^ COME-CCT was designed in a multicentric and multicontinental fashion according to a prespecified study protocol,^11^ and the COME-CCT IPD meta-analysis was registered at PROSPERO.

### Eligibility criteria and study selection

COME-CCT was designed as a prospective meta-analysis of IPD from prospective diagnostic accuracy studies comparing coronary CTA with invasive coronary angiography as the reference standard. Both tests used a diameter stenosis of at least 50% as the cutoff value to define angiographically obstructive CAD.

Eligible patients needed to have a clinical indication for coronary angiography due to suspected CAD because of stable chest pain, and both tests had to be performed in all patients to avoid verification bias.^15^ Results had to be provided using 2×2 or 3×2 cross tabulations for the comparison of CTA with coronary angiography.^16^ For the calculation of clinical pretest probability using an updated version of the Diamond and Forrester method,^17^ ^18^ the following information had to be provided: patient age, sex, and angina pectoris type.^19^ ^20^ Angina pectoris was classified as typical angina, atypical angina, non-anginal chest pain, or other chest discomfort according to Diamond and Forrester. The primary analysis included all patients irrespective of whether they had diagnostic or non-diagnostic (that is, unevaluable) CTA examinations.^16^

The search was performed in three databases (Medline, Embase, and Web of Science; sensitive search strategy described in web appendix 2).^21^ The search strategy was implemented for every database by two independent investigators of the central data management team, as described in the published protocol.^11^

### Collection and harmonisation of individual patient level data

When the search was completed, we started IPD collection and subsequent data harmonisation. As defined in the study protocol,^11^ we emailed corresponding authors of eligible published studies identified by the search with a cover letter detailing the objectives of the collaborative meta-analysis and a CD containing a uniform IPD collection file (web appendix 3).^11^ Other authors were contacted if the corresponding author did not respond. Data from unpublished studies that met the inclusion criteria were retrieved from corresponding authors of published studies.^22^ The completed IPD collection files were sent back to the data management team.

Data harmonisation was performed by two independent readers at the site of the central data management team, who analysed data and searched for non-plausible data, including range checks, average and median checks (*v* published aggregated data results), minimum and maximum checks (*v* aggregated data results), wrong entries, non-logical values, and other data checks. Aggregate data of studies for which IPD were not provided were collected and compared with aggregate data of studies for which IPD were provided to rule out study selection bias. Aggregate data consisted of all data necessary for 2×2 tabulations to estimate sensitivity, specificity, and PPVs and NPVs, and to perform receiver operating characteristic curves analysis. Risk of bias and applicability concerns of the included studies were assessed by two independent readers of the central data management team, who were not involved in data harmonisation, using the QUADAS-2 tool.^23^

### Primary and secondary outcomes

The primary outcomes of interest were the PPV and NPV of coronary CTA for the presence of obstructive CAD as measures of the post-test probability of disease needed for the no-treat/treat threshold model. PPV and NPV were evaluated as a function of the pretest probability of obstructive CAD and analysed by a generalised linear mixed model meta-regression including non-diagnostic CTA results.^11^ To define the range of appropriate pretest probabilities of obstructive CAD for CTA, we used the no-treat/treat threshold method.^12^ Following the European Society of Cardiology guidelines, the no-treat/treat thresholds for CTA were 15% and 50%, respectively, on the disease probability range. This means that for disease probabilities below 15%, other reasons for the chest pain should be considered, and for values above 50%, ischaemia testing should be recommended.^3^

Secondary outcomes were sensitivity and specificity analyses in women and men, in patients of different age groups, and with different angina pectoris types. Diagnostic performance of CTA was descriptively compared in computed tomography scanners with up to 64 detector rows versus those with more than 64 detector rows. A further post hoc analysis, which was not defined in the protocol of COME-CCT, were requested by reviewers: we analysed the use of core laboratories and quantitative coronary angiography in relation to diagnostic accuracy of CTA.

### Statistical analysis

Using an intention-to-diagnose approach, we implemented a worst case scenario in which non-diagnostic CTA results were considered false positive if coronary angiography was negative, and considered false negative if coronary angiography was positive.^16^ We calculated clinical pretest probability using the validated CAD Consortium prediction tool, which is an updated version of the Diamond and Forrester model.^17^ ^18^ Specifically, probability was estimated using all elements of the prediction tool: patient age, sex, and clinical presentation (angina pectoris type). Based on this model, mean logit PPVs and NPVs with their standard errors and 95% confidence intervals were estimated. These quantities were back-transformed to the original scale to obtain summary PPVs and NPVs. For the statistical analysis, we applied a univariate logistic regression model^24^ extended by incorporating a random effect for study and a random slope for CTA or coronary angiography results, which is equivalent to a bivariate generalised linear mixed model.^25^ To maintain equivalence, interaction terms of CTA and covariates of interest are necessary and were thus included into the model. Using these data and model, we performed a statistical prediction for a new cohort following the ideas presented by Skrondal and Rabe-Hasketh.^26^ ^27^

To apply the no-treat/treat CAD probability thresholds for CTA, we chose two post-test probabilities to define the range when to offer CTA: below 15% when other reasons for the chest pain should then be considered (no treat threshold) and probabilities above 50% when ischaemia testing is then recommended (treat threshold). We then calculated the clinical prediction score pretest probabilities that yielded post-test probabilities after negative CTA of below 15% (that is, a NPV of at least 85%) and those after positive CTA of above 50% (that is, a PPV of at least 50%). In addition to the model in which non-diagnostic CTA results were considered false positive if coronary angiography was negative and false negative if coronary angiography was positive, we also calculated NPVs and PPVs depending on pretest probabilities in a model excluding non-diagnostic CTA.

Based on the generalised linear mixed model with the test result as a dependent variable, we estimated mean logit sensitivity and specificity, between-study variability in logit sensitivity and specificity, and the covariance between them and the effect of covariates. Areas under the receiver operating characteristic curves were constructed using the observed data and model based predictions. These also included random effects, which reflect variability between studies and unobserved influential variables. The clinical performance of CTA was compared including non-diagnostic CTA results between women and men, between four age groups, and between the four angina pectoris types. Furthermore, it was compared with quantitative coronary angiography as the reference standard and the presence of core laboratories in the case of multicentre studies, to determine if this affected the primary outcomes. The influence of these covariates was evaluated by the likelihood ratio test. We did not search for the most parsimonious statistical model because, for clinical reasons, the type of chest pain, for instance, is pivotal and should be included. To compare the area-under-the-curve results for inclusion versus exclusion of non-diagnostic examinations, we applied DeLongs’ test.^28^ Performance of CTA using up to 64 or more than 64 detector rows was compared empirically. We investigated publication bias using the rank based method for the arcsine difference of study specific sensitivities and specificities.^29^

As recommended by the PRISMA statement, we compared aggregate data of studies for which IPD was provided with those aggregate data of studies for which IPD was not provided to identify or exclude differences between these data based on a bivariate generalised linear mixed model with IPD available as a covariate.^30^ We calculated the likelihood ratio test for the models with and without this covariate. This model was estimated for aggregate 2×2 tables using the model of Chu and Cole.^31^ In our analyses, non-diagnostic CTA results were treated using an intention-to-diagnose approach (see above) as suggested by Schuetz and colleagues.^16^ Estimation was done with Stata 14, using the packages GLLAMM and gllapred for the predictions and MIDAS for the 2×2 diagnostic meta-analysis. Further statistical analyses were conducted by SAS version 9.4 (SAS Institute, Cary, NC, USA) and R 3.4,^32^ and packages lme4,^33^ meta,^34^ and pROC.^35^ Model based estimates of sensitivity and specificity were obtained by averaging over the random effects using the R package lsmeans.^36^

### Patient and public involvement

We have insufficient evidence to comment on whether patients were actively involved in the design or management of the 76 studies included and for which IPD were provided. In this study, given the privacy concerns of IPD sharing, it was also not practical to involve patients in reviewing this data. The results of the study will be disseminated using press releases and the website of the study coordinator.

## Results

### Included studies and study participants

**Fig 1:**
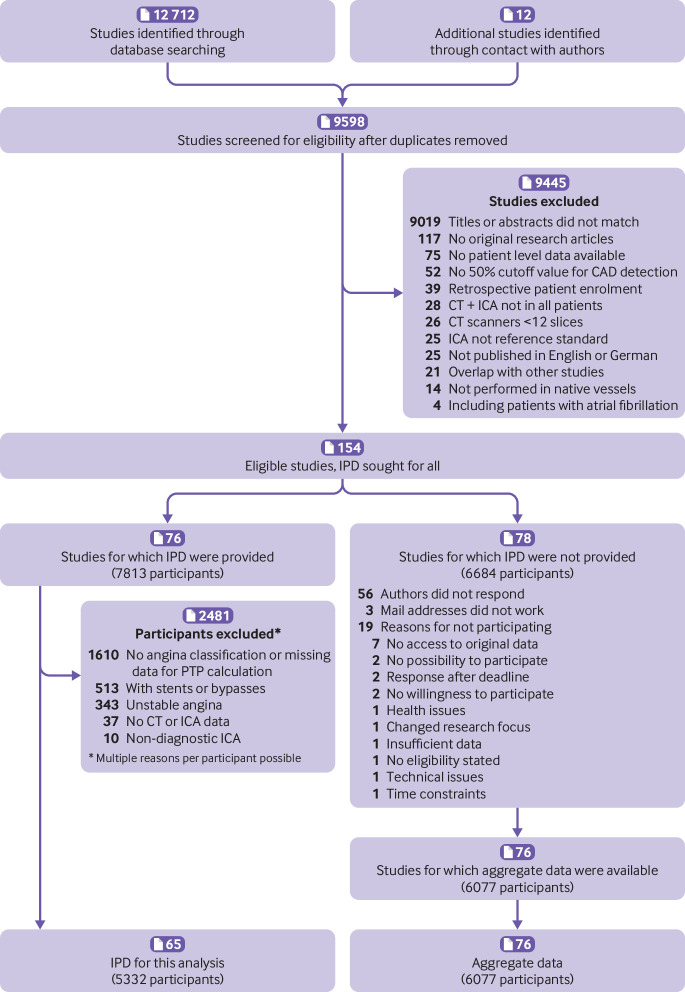
PRISMA individual patient data (IPD) flow diagram. A total of 9598 studies were scanned after removing duplicates. After full text review of 580 publications, 154 studies remained for which IPD were sought. IPD were retrieved for 76 studies including 7813 participants. For this analysis, 2481 participants of 11 studies had to be excluded, mainly because angina pectoris type was not classified or other data for pretest probability (PTP) calculation were missing (1610). Further reasons for exclusion of participants from the main analysis included coronary stents or bypass grafts, unstable angina pectoris, and non-diagnostic, invasive, coronary angiography examinations. A total of 5332 patients were included in this IPD analysis. ICA=invasive coronary angiography

We identified 154 eligible published studies, for which we sought IPD by contacting authors (fig 1). Overall, we identified 76 studies (74 published, 2 unpublished) for which IPD were provided for a total of 7813 participants. Of these 76 studies, 11 (including 2481 patients) had to be excluded from this main COME-CCT analysis because no information on angina pectoris presentation was recorded, chest pain was unstable, or patients had coronary artery stents or bypasses. Finally, a total of 5332 patients from the remaining 65 studies (63 published and two unpublished; supplementary table 1 in web appendix 2) were included in the main collaborative analysis, including 554 patients with non-diagnostic CTA examinations (fig 1). Risk of bias was low for all items in most studies, and applicability concerns were not present in any of the included studies assessed by the QUADAS-2 tool (supplementary tables 2-4 and supplementary figures 1-2 in web appendix 2). In most of the 78 studies for which IPD were not provided (for a total of 6684 patients included), the corresponding authors did not respond (56/78, 72%) and aggregate data were available for 76 studies, or 6077 patients (fig 1).

### Patient characteristics

Patient characteristics of the 5332 patients from 64 studies available for IPD analysis and their assignment to pretest probability categories are presented in table 1. Patient characteristics for each dataset including chest pain symptoms and risk factor distribution are listed in supplementary table 5 in web appendix 2. Technical characteristics of imaging tests for each dataset are summarised in supplementary table 6 in web appendix 2. Table 1 shows empirical results for true positives and negatives, false positives and negatives, as well as non-diagnostic CTA results for different categories of clinical pretest probability. Up to a pretest probability of 40%, pretest probability predictions overestimated true CAD prevalence by about 10 percentage points using the updated version of the Diamond and Forrester method. Above a pretest probability of 50%, true CAD prevalence was underestimated by about 10 percentage points. Above a pretest probability of about 70%, empirical diagnostic accuracy including specificity decreased (table 1). Also, CTA using up to 64 versus more than 64 detector rows led to significantly lower empirical sensitivity (86.5% *v* 93.4%, P=0.002) and specificity (72.6% *v* 84.4%, P<0.001, table 1). Non-diagnostic examinations were rare for scanners with more than 64 detector rows (2.9%), but considerably more frequent on those with up to 64 detector rows (11.5%, table 1).

**Table 1:** Baseline patient characteristics and empirical diagnostic performance of computed tomography angiography to diagnose coronary artery disease, stratified by pretest probability category and scanner detector rows

	Overall (n=5332)	Pretest probability categories									
		0 to <10% (n=86)	10 to <20% (n=530)	20 to <30% (n=601)	30 to <40% (n=727)	40 to <50% (n=745)	50 to <60% (n=752)	60 to <70% (n=590)	70 to <80% (n=535)	80 to <90% (n=698)	90 to 100% (n=68)
**Demographic characteristics (median (range) or No (%))**											
Median age (years)	61 (18-96)	47 (18-50)	56 (23-70)	59 (24-82)	57 (37-89)	55 (27-87)	63 (30-91)	70 (36-88)	55 (47-92)	66 (59-77)	80 (78-89)
Men	3473 (65)	0	29 (5)	211 (35)	509 (70)	576 (77)	507 (67)	391 (66)	484 (90)	698 (100)	68 (100)
Women	1859 (35)	86 (100)	501 (95)	390 (65)	218 (30)	169 (23)	245 (33)	199 (34)	51 (10)	0	0
Median body mass index	26.3 (14.3-57.1)	25.6 (17.9-39.3)	26.2 (14.3-47.3)	25.9 (16.1-44.8)	26.2 (16.9-41.8)	26.4 (17.5-45.2)	26.5 (17.2-57.1)	26.4 (15.5-56.2)	27.0 (17.5-42.5)	26.9 (16.9-56.7)	25.9 (16.8-35.2)
**Clinical presentation (No)**											
Typical angina	1967	0	0	4	43	137	247	306	464	698	68
Atypical angina	1592	1	138	269	235	280	339	260	70	0	0
Non-anginal chest pain	796	38	162	157	188	158	80	12	1	0	0
Other chest discomfort	977	47	230	171	261	170	86	12	0	0	0
**Diagnostic performance (No or %)**											
CAD prevalence (%)*	48.3	17.4	24.0	32.1	40.9	46.8	46.8	53.7	68.6	71.6	82.4
TP	2251	14	120	176	272	321	310	256	317	420	45
TN	2031	52	313	312	334	287	294	194	103	134	8
FP	728	19	90	96	96	109	106	79	65	64	4
FN	322	1	7	17	25	28	42	61	50	80	11
NDX†	554	13	58	50	54	67	76	79	60	85	12
NDX rate (%)†	10.4	15.1	10.9	8.3	7.4	9.0	10.1	13.4	11.2	12.2	17.6
PPV (%)	75.6	42.4	57.1	64.7	73.9	74.7	74.5	76.4	83.0	86.8	91.8
NPV (%)	86.3	98.1	97.8	94.8	93.0	91.1	87.5	76.1	67.3	62.6	42.1
Sensitivity (%)	87.5	93.3	94.5	91.2	91.6	92.0	88.1	80.8	86.4	84.0	80.4
Specificity (%)	73.6	73.2	77.7	76.5	77.7	72.5	73.5	71.1	61.3	67.7	66.7
Diagnostic accuracy (%)	80.3	76.7	81.7	81.2	83.4	81.6	80.3	76.3	78.5	79.4	77.9
LR+	3.32	3.49	4.23	3.88	4.10	3.34	3.32	2.79	2.23	2.60	2.41
LR−	0.17	0.09	0.07	0.12	0.11	0.11	0.16	0.27	0.22	0.24	0.29
**CT scanners with ≤64 detector rows (No or %)**											
No of patients	4666	80	452	529	651	634	637	530	472	619	62
CAD prevalence (%)	48.2	17.5	24.1	31.2	41.8	45.5	46.0	54.0	67.4	72.5	85.5
TP	1943	13	102	150	248	264	256	226	270	372	42
TN	1757	47	265	273	295	247	246	170	95	114	5
FP	662	19	78	91	84	99	98	74	59	56	4
FN	304	1	7	15	24	24	37	60	48	77	11
NDX†	538	13	54	50	50	65	75	77	58	84	12
NDX rate (%)†	11.5	16.3	11.9	9.5	7.7	10.3	11.8	14.5	12.3	13.6	19.4
PPV (%)	74.6	40.6	56.7	62.2	74.7	72.7	72.3	75.3	82.1	86.9	91.3
NPV (%)	85.2	97.9	97.4	94.8	92.5	91.1	86.9	79.9	66.4	59.7	31.3
Sensitivity (%)	86.5	92.9	93.6	90.9	91.2	91.7	87.4	79.0	84.9	82.9	79.2
Specificity (%)	72.6	71.2	77.3	75.0	77.8	71.4	71.5	69.7	61.7	67.1	55.6
Diagnostic accuracy (%)	79.3	75.0	81.2	80.0	83.4	80.6	78.8	74.7	77.3	78.5	75.8
LR+	3.16	3.23	4.12	3.64	4.11	3.20	3.07	2.61	2.22	2.52	1.78
LR−	0.19	0.10	0.08	0.12	0.11	0.12	0.18	0.30	0.24	0.26	0.37
**CT scanners with >64 detector rows (No or %)**											
No of patients	558	6	73	62	66	87	103	55	41	59	6
CAD prevalence (%)*	46.1	16.7	21.9	37.1	30.3	54.0	49.5	50.9	75.6	62.7	50.0
TP	240	1	16	21	19	44	46	27	29	34	3
TN	254	5	46	36	39	33	47	22	6	17	3
FP	47	0	11	3	7	7	5	5	4	5	0
FN	17	0	0	2	1	3	5	1	2	3	0
NDX†	16	0	4	0	4	2	1	2	2	1	0
NDX rate (%)†	2.9	0.0	5.5	0.0	6.1	2.3	1.0	3.6	4.9	1.7	0.0
PPV (%)	83.6	100	59.3	87.5	73.1	86.3	90.2	84.4	87.9	87.2	100
NPV (%)	93.7	100	100	94.7	97.5	91.7	90.4	95.7	75.0	85.0	100
Sensitivity (%)	93.4	100	100	91.3	95.0	93.6	90.2	96.4	93.5	91.9	100
Specificity (%)	84.4	100	80.7	92.3	84.8	82.5	90.4	81.5	60.0	77.3	100
Diagnostic accuracy (%)	88.5	100	84.9	91.9	87.9	88.5	90.3	89.1	85.4	86.4	100
LR+	5.98	∞	5.18	11.87	6.24	5.35	9.38	5.21	2.34	4.04	∞
LR−	0.08	0	0	0.09	0.06	0.08	0.11	0.04	0.11	0.10	0

TP=true positives; TN=true negatives; FP=false positives; FN=false negatives; PPV=positive predictive value; NPV=negative predictive value; LR+=positive likelihood ratio; LR−=negative likelihood ratio; NDX=non-diagnostic results.

The empirical results were derived from raw data and thus differ from the results of the statistical model.

* CAD prevalence was defined by coronary angiography.

† Non-diagnostic results were included in the estimation of diagnostic accuracy as false positives if the reference standard was negative, and as false negative if the reference standard was positive.

### Diagnostic performance of CTA depending on pretest probability

**Fig 2:**
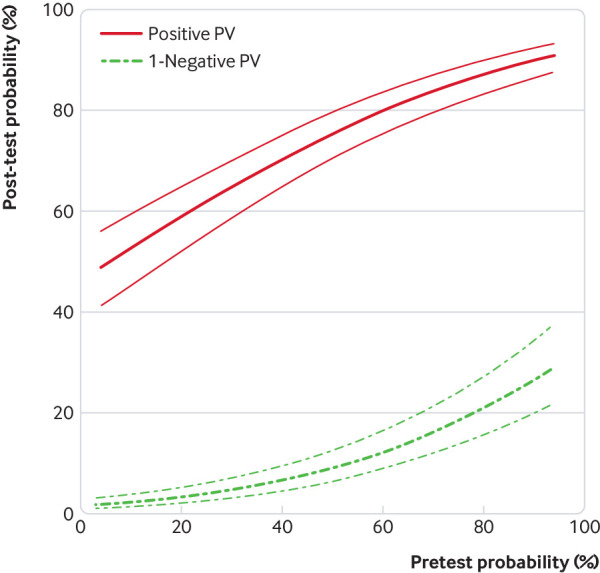
Clinical diagnostic performance of computed tomography angiography to diagnose obstructive coronary artery disease as a function of pretest probability. The x axis represents the predicted clinical pretest probability, and the y axis shows the post-test probabilities and thus the positive predictive value (PV) and 1−negative PV with their 95% confidence intervals, based on the generalised linear mixed model including non-diagnostic CTA examinations. Results for the generalised linear mixed model excluding non-diagnostic CTA examinations are shown in supplementary figure 3 in web appendix 2. Disease probabilities were predicted by averaging over the random effects distribution. AUC=area under the curve

Figure 2 shows the relation between PPVs and NPVs of CTA and clinical pretest probability in the generalised linear mixed model including non-diagnostic examinations. Based on the statistical model, we found that for a pretest probability range of 7-67%, the treat threshold of more than 50% post-test probability (ischaemia testing recommended) and the no-treat threshold of less than 15% (consider other reasons for the chest pain) were obtained using CTA. At a pretest probability of 7%, the PPV of CTA was 50.9% (43.3% to 57.7%) and NPV was 97.8% (96.4% to 98.7%). At a pretest probability of 67%, the PPV was 82.7% (78.3% to 86.2%) and NPV was 85.0% (80.2% to 88.9%).

When excluding non-diagnostic examinations, the PPV at a pretest probability of 7% was 68.0% (60.5% to 74.6%) and NPV was 98.3% (97.0% to 99.1%; table 2); at a pretest probability of 67%, the PPV was 88.9% (85.3% to 91.7%) and NPV was 91.4% (87.8% to 94.2%). The relation between PPVs and NPVs of CTA and clinical pretest probability in the generalised linear mixed model after excluding non-diagnostic CTA examinations is shown in supplementary figure 3 in web appendix 2. The model based predictive values for 7% and 67% pretest probabilities as well as for 15% and 50% pretest probabilities as recommended by the European Society of Cardiology guidelines are listed in table 2 for both, including and excluding non-diagnostic CTA results.

**Table 2:** Model based predictive values of computed tomography angiography for obstructive coronary artery disease, including and excluding non-diagnostic results

	Pretest probability of coronary artery disease (%)			
	7	15	50	67
**Including non-diagnostic examinations**				
PPV (%; 95% CI)	50.9 (43.3 to 57.7)	55.8 (48.6 to 62.3)	75.4 (70.5 to 79.5)	82.7 (78.3 to 86.2)
NPV (%; 95% CI)	97.8 (96.4 to 98.7)	97.1 (95.4 to 98.2)	90.9 (87.5 to 93.4)	85.0 (80.2 to 88.9)
**Excluding non-diagnostic examinations**				
PPV (%; 95% CI)	68.0 (60.5 to 74.6)	71.6 (64.7 to 77.5)	84.5 (80.0 to 87.9)	88.9 (85.3 to 91.7)
NPV (%; 95% CI)	98.3 (97.0 to 99.1)	97.9 (96.4 to 98.8)	94.4 (92.0 to 96.3)	91.4 (87.8 to 94.2)

PPV=positive predictive value, NPV=negative predictive value.

### Clinically important subgroups

The sensitivity of CTA for all patients was 95.2% (92.6% to 96.9%) and the specificity was 79.2% (74.9% to 82.9%, table 3). The sensitivity of CTA for women and men was 93.5% (89.6% to 96.0%) and 95.8% (93.4% to 97.4%), respectively, while the specificity was 80.6% (75.9% to 84.6%) and 77.4% (72.4% to 81.8%, likelihood ratio test****11.28, df: 2, P<0.001, table 3). Empirical data of women and men and their assignment to pretest probability categories are tabulated in supplementary tables 7 and 8 in web appendix 2. For patients older than 75, the sensitivity of CTA was 93.2% (88.6% to 96.0%) and the specificity was 73.6% (65.7% to 80.2%, table 3).

**Table 3:** Model based sensitivity and specificity of computed tomography angiography for obstructive coronary artery disease, according to total population and subgroups

	Diagnostic performance estimate				
	Sensitivity			Specificity	
	Estimate (SE)	95% CI		Estimate (SE)	95% CI
Total	95.2 (1.1)	92.6 to 96.9		79.2 (2.1)	74.9 to 82.9
Sex					
Women	93.5 (1.6)	89.6 to 96.0		80.6 (2.2)	75.9 to 84.6
Men	95.8 (1.0)	93.4 to 97.4		77.4 (2.4)	72.4 to 81.8
Age					
>75	93.2 (1.8)	88.6 to 96.0		73.6 (3.7)	65.7 to 80.2
>65 to ≤75	95.0 (1.2)	92.0 to 96.9		77.3 (2.6)	71.8 to 82.0
>50 to ≤65	95.1 (1.2)	92.3 to 97.0		80.6 (2.1)	76.1 to 84.5
≤50	95.5 (1.4)	91.8 to 97.6		83.8 (2.4)	78.6 to 87.9

SE=standard error.

**Fig 3:**
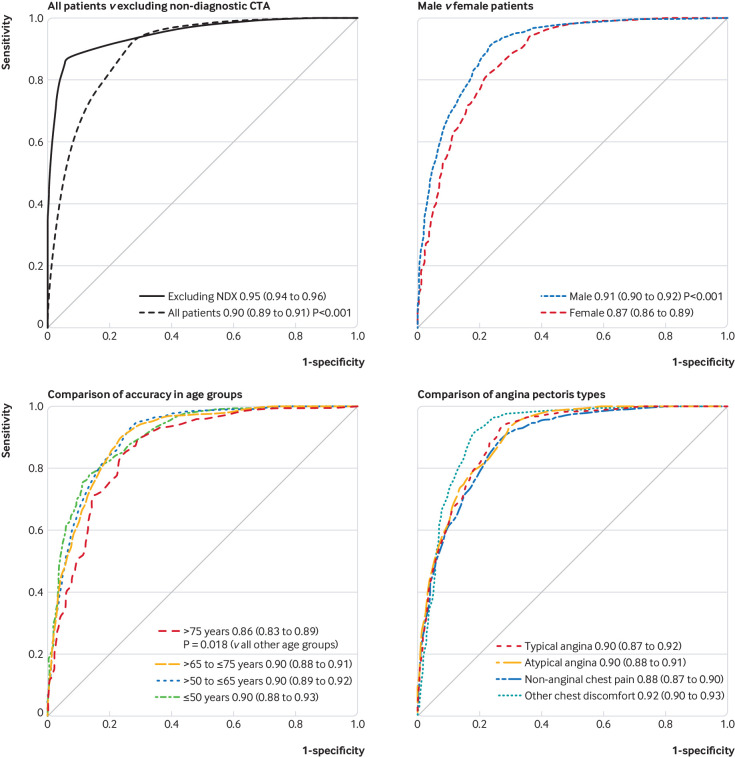
Receiver operating characteristic curves of computed tomography angiography for obstructive coronary artery disease, by subgroup and after excluding non-diagnostic examinations (NDX). Diagnostic performance results are shown for all patients versus results obtained after exclusion of non-diagnostic test results. The inclusion of all patients (top left panel) resulted in lower performance, which is a more accurate prediction of the real world performance to be expected. Thus, all subgroup comparisons in the other three panels are provided for all patients (including non-diagnostic examinations): diagnostic performance was higher in men and lower in patients older than 75, and angina pectoris types were not significantly associated with performance. Curves were generated by a generalised linear mixed model and predictions based on these models. Computations were performed with the statistical package R and packages lme4 and pROC. Areas under the curve were constructed by use of the observed data and model based predictions, which also included the random effects reflecting variability between studies and unobserved influential variables

In the receiver operating characteristic analysis, CTA including non-diagnostic results had an area under the curve of 0.897 (95% confidence interval 0.889 to 0.906) versus a significantly larger area under the curve when non-diagnostic results were excluded (0.949 (0.943 to 0.954), P<0.001, fig 3). All further results are provided for the analyses with inclusion of non-diagnostic CTA examinations. The diagnostic performance of CTA was lower in women than in men (area under the curve 0.874 (0.858 to 0.890) *v* 0.907 (0.897 to 0.916), P<0.001, fig 3). A descriptive analysis revealed that the heart rate during CTA was higher in women than in men (supplementary figure 4 in web appendix 2). Heart rate was the only factor significantly associated with a non-diagnostic CTA examination (supplementary table 9 in web appendix 2). The lowest diagnostic performance of CTA was found in patients older than 75 (0.864, 0.834 to 0.894, P=0.018 *v* all other age groups, fig 3). Empirical data of patients of different age groups and their assignment to pretest probability categories are listed in supplementary tables 10-13 in web appendix 2. In a descriptive analysis, patients older than 75 had significantly higher coronary artery calcium scores****than younger patients (supplementary figure 5 in web appendix 2).^37^ Accuracy of CTA was not significantly influenced by the angina pectoris type (area under the curve for typical angina 0.895 (0.873 to 0.917) *v* atypical angina 0.898 (0.884 to 0.913) *v* non-anginal chest pain 0.884 (0.870 to 0.899) *v* other chest discomfort: 0.915 (0.897 to 0.934), fig 3).

### Further post hoc analyses

Empirical data of patients with different chest pain types and their assignment to pretest probability categories are tabulated in supplementary tables 14-17 in web appendix 2. The receiver operating characteristic analysis after excluding non-diagnostic CTA results showed that accuracy was significantly reduced only in patients older than 75, whereas sex was no longer a significant factor (supplementary figure 6 in web appendix 2). Overall, 3615 (69%) of 5266 patients were analysed with quantitative coronary angiography as the reference standard. We found no significant difference in diagnostic accuracy of CTA, irrespective of whether quantitative coronary angiography was used or not, while the use of core laboratories was associated with lower sensitivity and specificity (supplementary table 18).

### Participation and publication bias

**Fig 4:**
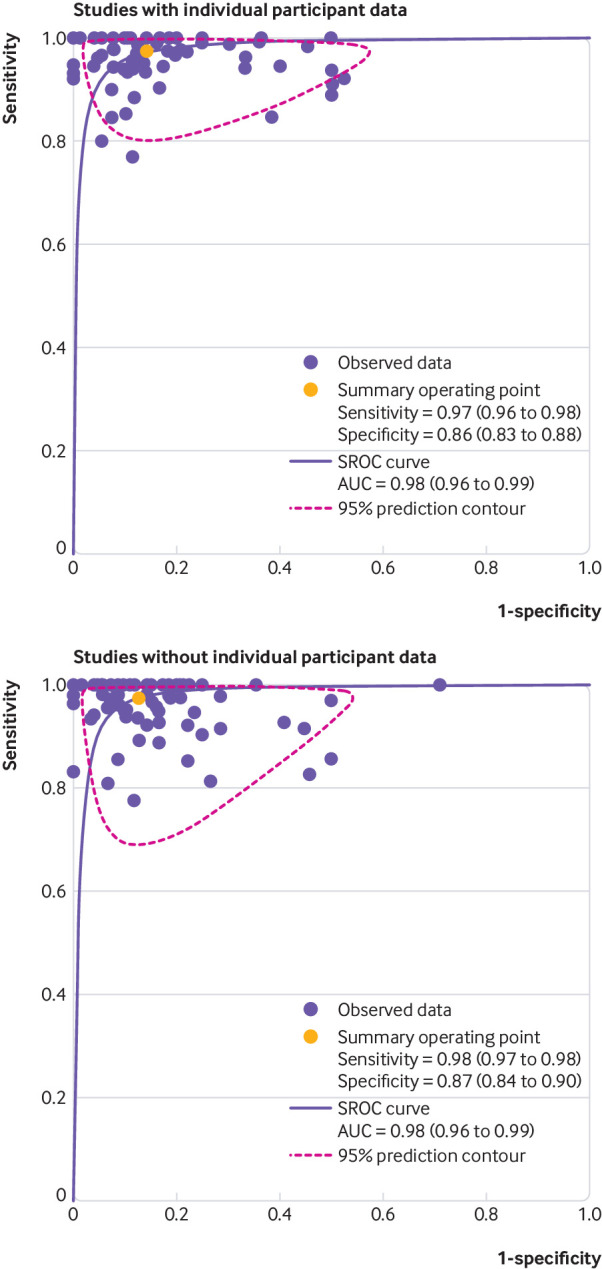
Summary receiver operating characteristic (SROC) curves for studies using computed tomography angiography to diagnose obstructive coronary artery disease, with and without individual participant data (IPD) available. Curves are shown for studies with IPD available versus studies for which no IPD were available. Curves were calculated by aggregated data methodology (SROC curves) both for panels and after excluding non-diagnostic test results, which were not consistently available in publications of studies that did not provide IPD. Of 76 studies that provided IPD, aggregate data were not available for seven studies (two unpublished), leaving 69 for the analysis of studies with IPD; of 78 studies that did not provide IPD, 76 had aggregate data available (fig 1); there was no significant difference in diagnostic performance between these two groups of diagnostic accuracy studies (P=0.73). Further details shown in table 4. For study number details, see supplementary figure 8. AUC=area under the curve

The comparison of the diagnostic accuracy studies for which IPD were provided with those studies for which only aggregate data were available (fig 1) showed no significant difference in diagnostic performance (P=0.73, fig 4 and table 4). We found no publication bias (supplementary figure 7 in web appendix 2), and heterogeneity analysis yielded variances of random effects of 0.673 for 1−specificity and 3.667 for sensitivity (table 5). We obtained similar values after adjusting age, sex, and type of chest pain (table 6), indicating that these covariates do not explain heterogeneity between studies. Table 6 also presents the model coefficients used for generating the receiver operating characteristic curves.

**Table 4:** Study specific diagnostic accuracy of computed tomography angiography for coronary artery disease, comparing aggregated data from studies with individual participant data (IPD) versus aggregated data from studies without IPD

	Estimate (95% CI)	
	Sensitivity	Specificity
All studies	0.97 (0.97 to 0.98)	0.87 (0.85 to 0.88)
Studies with IPD*	0.97 (0.96 to 0.98)	0.86 (0.83 to 0.88)
Studies without IPD*	0.98 (0.97 to 0.98)	0.87 (0.84 to 0.90)
Heterogeneity analysis, IPD=1	Likelihood ratio test, χ^2^=0.62	P=0.73

* Studies with IPD=studies for which IPD were provided; studies without IPD=studies for which IPD were not provided. There was no relevant difference between these two groups of studies.

**Table 5:** Heterogeneity analysis of diagnostic accuracy studies using computed tomography angiography to diagnose obstructive coronary artery disease: overall statistical model without covariates

	Generalised linear mixed model fit by maximum likelihood
Fixed effects*	
Intercept	Estimate (SE), −1.336 (0.125); z=−10.72; P<0.001
CAD present	Estimate (SE), 4.313 (0.294); z=14.69; P<0.001
**Random effects†**	
Study No (intercept)	Variance, 0.673; SD, 0.8203
CAD present	Variance, 3.667; SD, 1.9149; correlation, −0.75

SE=standard error; SD=standard deviation; CAD=coronary artery disease.

* Data for fixed effects are estimates (standard error) of regression coefficients, z value, and P value. The intercept is 1−specificity, and the sum of the intercept and CAD represents sensitivity.

† Data are for random effects are variance, standard deviation, and correlation. Random effects quantify the variability between studies. The variance of the random effects of the intercept corresponds to the between study variability of 1−specificity, and the random effects variance of CAD denotes the between study variability of sensitivity.

**Table 6:** Heterogeneity analysis of diagnostic accuracy studies using computed tomography angiography to diagnose obstructive coronary artery disease: analysis of potential effects of covariates in statistical model

Covariates	Generalised linear mixed model fit by maximum likelihood		
	Estimate (SE)	z value	P value
Fixed effects*			
Intercept	−1.566 (0.218)	−7.188	<0.001
CAD present	4.401 (0.492)	8.944	<0.001
Male sex	0.194 (0.096)	2.027	<0.05
Typical angina	−0.303 (0.192)	−1.579	0.11
Atypical angina	−0.192 (0.170)	−1.125	0.26
Non-anginal chest pain	−0.196 (0.175)	−1.116	0.26
Age			
50-65	0.215 (0.140)	1.538	0.12
65-75	0.417 (0.154)	2.716	0.01
>75	0.618 (0.198)	3.117	0.002
CAD present†			
+Sex	0.265 (0.187)	1.417	0.16
+Typical angina	0.494 (0.399)	1.237	0.22
+Atypical angina	−0.021 (0.359)	−0.059	0.95
+Non-anginal	0.153 (0.349)	0.438	0.66
+Age >50 to ≤65	−0.290 (0.285)	−1.016	0.31
+Age >65 to ≤75	−0.517 (0.302)	−1.708	0.09
+Age >75	−1.055 (0.356)	−2.966	0.003
**Random effects‡**			
Study No (intercept)	Variance, 0.703	SD, 0.838	—
CAD present	Variance, 3.802	SD, 1.950	Correlation, −0.77

SE=standard error; SD=standard deviation; CAD=coronary artery disease.

* Data for fixed effects are estimates (standard error) of regression coefficients, z value, and P value.

† The variable “CAD present” describes the invasive coronary angiography result (1=positive). “+Sex” describes the interaction between the invasive coronary angiography results and sex, and so on. These interactions are needed to maintain the bivariate structure of the diagnostic accuracy data.

‡ Rather than estimates (standard error) of regression coefficients, z value, and P value, data for random effects are variance, standard deviation, and correlation, respectively. The variance of the random effects quantifies the variability between studies for sensitivity and specificity. The variance of the random effects of the intercept corresponds to the between study variability of 1−specificity and the random effects variance of CAD present to between study variability of sensitivity.

## Discussion

In this pooled analysis of patient level data, we show that coronary CTA is most appropriately implemented for clinical decision making in patients with suspected obstructive CAD and a pretest probability ranging from 7% to 67%. In this low-to-intermediate clinical probability range, coronary CTA was able to accurately stratify patients into those with a disease post-test probability of below 15%, in whom other reasons for the chest pain should be considered, and those with a probability above 50%, in whom further testing is recommended.^3^

Our study also showed that the diagnostic performance of CTA was not significantly influenced by the angina pectoris type, but it was higher in men and lower in older patients. After we excluded non-diagnostic examinations from the analysis, the accuracy of CTA improved and the difference in diagnostic performance between female and male patients became non-significant. Moreover, diagnostic examinations are now more commonly conducted by computed tomography scanners with more than 64 detector rows, which had lower rates of non-diagnostic examinations.

### Clinical context and guidelines

Current European and US guidelines recommend calculating patients’ pretest probability of CAD to guide diagnostic decisions.^3^ ^38^ The European Society of Cardiology specifically recommends considering CTA in patients with 15-50% pretest probability of obstructive CAD,^3^ whereas the NICE guideline recommends coronary CTA as the primary imaging test for all patients with possible angina and suspected obstructive CAD.^4^ Our results show that using the no-treat/treat threshold approach, CTA offers good to excellent results in pretest probability range of 7% to 67%. The procedure yields a post-test probability below 15%, where other reasons for the chest pain should be considered, in case of negative CTA (that is, NPV ≥85%); and above 50%, where ischaemia testing is recommended, in case of positive CTA (that is, PPV ≥50%). Since no IPD meta-analysis has so far investigated in which patients CTA has the highest diagnostic performance, the results presented here might have important implications for current guidelines. The results of the diagnostic performance model can also be used to define the appropriate pretest probability range depending on the NPV and PPV deemed to be acceptable for the specific diagnostic purpose.

The main clinical strength of coronary CTA is its high NPV, and this is supported by our findings, which show that CTA can also detect both obstructive and non-obstructive CAD and therefore is a suitable imaging modality to guide subsequent management.^39^ This may make patient management more efficient and can also lower costs, not least by reducing the high rate of negative coronary angiographies performed annually. Recently published randomised clinical trials support these assumptions. Although the PROMISE trial—which compared CTA with an initial functional testing strategy in the evaluation of chest pain—did not show a reduction in major adverse cardiovascular events (defined as death, myocardial infarction, and unstable angina needing hospital admissions, or a major procedural complication), subsequent invasive coronary angiography was more effective in the CTA group.^40^

The SCOT-HEART trial prospectively compared standard care with standard care plus CTA for the diagnosis of CAD in patients with recent onset chest pain.^41^ In the trial, CTA was found to increase diagnostic certainty, increase the identification of obstructive and non-obstructive CAD, and eliminate the need for further downstream stress imaging tests.^41^ Furthermore, the five year clinical outcome analysis of SCOT-HEART showed that standard care plus CTA resulted in a halving of fatal and non-fatal myocardial infarction without increasing the five year rate of coronary revascularisations but initiating more targeted preventive and anti-anginal treatments.^9^ However, some controversy remains about the use of coronary CTA as the first line diagnostic test in patients with stable chest pain and suspected CAD,^42^ and our IPD meta-analysis provides insights about in which patients CTA has highest predictive values.

Our IPD meta-analysis data can thus help physicians in better identifying the patients for whom coronary CTA is the most appropriate diagnostic test. Whether CTA can further improve clinical effectiveness in patients with a clinical indication for coronary angiography is an important question. The CAD-Man study showed that coronary CTA can reduce the need for invasive coronary angiography by up to 80% and can reduce procedural complications.^8^ A similar safety profile with non-inferiority of CTA versus invasive coronary angiography in terms of major cardiovascular events at one year was found in the CONSERVE trial.^43^ However, coronary CTA still has to be analysed in a multicentre study of patients with a clinical indication for invasive coronary angiography, and the randomised DISCHARGE trial will provide more data in this regard.^44^

### Comparison with other studies

Meta-analyses using aggregated data from studies that mostly excluded patients with non-diagnostic CTA examinations or considered them positive have reported a mean sensitivity for CTA per patient of 97.2% to 100% and a specificity of 87.4% to 89%.^21^ ^45^ We found lower sensitivities and specificities when including non-diagnostic tests as false positives or negatives in our IPD analysis in a worst case scenario, confirming that the performance of diagnostic tests is lower when non-diagnostic test results are considered and not merely excluded from the analysis.^16^ Our data also confirm the findings of a study level meta-regression analysis suggesting a hyperbolic decrease and increase of the NPVs and PPVs with increasing pretest probability, respectively.^7^ We also showed that pretest probability overestimated true CAD prevalence by about 10 percentage points up to a pretest probability of 40%; while above a pretest probability of 50%, true CAD prevalence was underestimated by about 10 percentage points. Future trials should address how to improve the accuracy of pretest probability estimation in patients with suspected CAD. Also, CTA using more than 64 detector rows led to significantly higher empirical sensitivity and specificity, indicating that recent CTA technology with more than 64 rows should be used.

Criteria have been proposed to ensure a reasonable use of coronary CTA.^46^ ^47^ Our study can help refine these criteria by allowing to individually define the appropriateness of coronary CTA based on the patient’s clinical pretest probability. Moreover, according to our findings, one should be cautious to use CTA in patients with a clinical pretest probability exceeding 67% since the NPV drops below 85%. In addition, the odds to find obstructive CAD on CTA (and thus also the likelihood to require another invasive test after non-invasive CTA) increases with the pretest probability. On the other hand, the PPV of coronary CTA becomes rather low in patients with a pretest probability of less than 7%, so that, in this situation, about half of the positive CTA examinations would result in unnecessary further testing. For ease of understanding, we visualised the predictive values of coronary CTA depending on pretest probability in figure 2. The European Society of Cardiology guidelines suggest a pretest probability range of 15-50% for diagnostic testing with coronary CTA. In this narrower range of pretest probability, CTA had an NPV and PPV of at least 90.9% and 55.8%, respectively.

From a clinical perspective, the diagnostic performance of CTA was not influenced by the angina pectoris type and was equally effective in ruling out angiographic CAD in patients with different angina pectoris types. Even though the reductions in diagnostic performance of CTA were small, decision makers should be aware that CTA has a slightly lower accuracy in patients older than 75, and in women compared with men, if non-diagnostic CTA results are included in the analysis. As mentioned above, non-diagnostic examinations are rarely seen when using computed tomography scanners with more than 64 detector rows; and when excluding non-diagnostic examinations, performance of CTA was similar in women and men. Similarly, our results showed that women had higher heart rates than men when examined by CTA and higher rates were the only factor associated with non-diagnostic examinations. Similar diagnostic accuracy of coronary CTA in men and women was reported by a multicentre study including 291 patients^48^ and by two single centre studies including 570 and 1372 patients.^49^ ^50^ In our IPD analysis of 3473 men and 1859 women including non-diagnostic examinations, we showed a small reduction in the area under the curve of CTA in women by 0.023 compared with men (fig 3). This difference might be explained by women being more likely to have high heart rates during CTA, which was the only factor significantly associated with non-diagnostic CTA results.

### Strength and limitations of study

Our study had strengths and limitations. IPD meta-analyses are considered the gold standard of systematic reviews. Even though the individual diagnostic accuracy studies were similar in terms of inclusion criteria and reference standard definitions, they varied in geographical origin and composition. Although this study was done in 22 countries and has a multicentric and multicontinental design, participation was not equally distributed across the globe, and ethnicity was not collected in data analysis. Moreover, obstructive CAD was defined by invasive coronary angiography as angiographically significant CAD in all patients, quantitative analysis of invasive angiography was used in 69% of patients, and functional definitions of CAD (eg, including invasive fractional flow reserve) were not used in the original studies. Thus, findings might not be generalisable to real world practice, although additional invasive fractional flow reserve is used in less than 10% of examinations worldwide,^51^ making the findings relevant for current clinical practice.

To define no-treat and treat thresholds, we estimated pretest probabilities by using the updated Diamond and Forester model (also recommended by the current the European Society of Cardiology guidelines). This calculator is validated for patients with suspected CAD referred for invasive coronary angiography, which is also the setting of this analysis. Other prediction models for pretest probabilities do not focus on this cohort but on patients referred for non-invasive assessment, as in the CONFIRM study.^52^ Furthermore, although results of exercise tests can also be included in pretest calculation, they are not included in currently validated probability calculators and could thus not be considered in our review.^53^

As shown in table 1, the most frequently used computed tomography scanners had 64 detector rows (2438 of 5332 patients); thus, CTA performance in clinical practice using state-of-the-art technology with more than 64 detector rows could have been even better. An important limitation of our IPD analysis of the clinical performance of coronary CTA was that not all 154 studies that were identified through our search strategy could be included because the responsible corresponding authors did not provide IPD. However, we sought to systematically retrieve all IPD from the studies identified by the systematic review and, despite several reminders, a relevant proportion of authors did not reply at all (56/154, 36%) or indicated that they could not participate in the COME-CCT Consortium because they had no access to original data (7/154, 5%). According to a systematic review of data retrieval in IPD meta-analyses, 68% of meta-analyses retrieved IPD from at least 80% of a median of only 14 eligible studies.^54^ With 154 eligible studies, our study was relevantly larger, which has been shown to complicate retrieval.^54^

Diagnostic performance results were similar in studies for which IPD were available versus those for which no IPD were provided. To include unpublished grey literature, we systematically asked all corresponding authors of the identified published studies about further unpublished analyses and systematically searched clinicaltrials.gov for unpublished diagnostic accuracy studies of coronary CTA and invasive coronary angiography registered in this database. With this approach, we found two unpublished studies that could be included in the COME-CCT database. Our findings did not show evidence of publication bias, but we found heterogeneity between studies, pointing to potentially unknown site specific factors that might have influenced diagnostic accuracy. All studies included patients who had suspected CAD and were clinically indicated to undergo coronary angiography. This gave us the opportunity to compare results from research CTA with clinically indicated coronary angiography in all patients to avoid verification bias. But the results are representative for patients clinically referred for coronary angiography, and there was likely to be bias particularly at the extremes of pretest probability. For instance, individuals with low pretest probability were likely to have other unmeasured risk factors that increased their clinical probability, which could have overestimated PPVs of CTA.

### Conclusions

In a no-treat/treat threshold model, the diagnosis of obstructive CAD using coronary CTA in patients with stable chest pain was most accurate when the clinical pretest probability was between 7% and 67%. Performance of CTA was not influenced by the angina pectoris type, was slightly higher in men, and was lower in older patients.

**What is already known on this topic**

Coronary computed tomography angiography (CTA) is an accurate non-invasive alternative to invasive coronary angiography, and can rule out coronary artery disease (CAD) with high certainty
By contrast with recent guidelines from the National Institute for Health and Care Excellence, the European Society of Cardiology recommends not considering CTA in all patients with typical and atypical angina, but only in patients with a 15-50% pretest probability of CAD, estimated by clinical information such as sex, age, and chest pain type

**What this study adds**

According to a no-treat/treat threshold model, patients with a pretest probability of CAD ranging from 7% to 67% could benefit most from coronary CTA to rule out or confirm CAD
CTA using more than 64 detector rows was empirically more sensitive and specific than CTA using up to 64 detector rows
Performance of CTA was not influenced by the angina pectoris type and was slightly higher in men and lower in older patients

## Web extra

Extra material supplied by authors

**Web appendix 1:** PRISMA statement for IPD systematic reviews (PRISMA-IPD) (page numbers refer to accepted manuscript)

**Web appendix 2:** Supplementary materials

**Web appendix 3:** IPD collection file

## Notes

Contributors: The COME-CCT investigators shared data, approved the manuscript after reviewing the findings and interpretation, provided critical revisions of the manuscript for important intellectual content, and gave final approval of the version to be published; and are accountable for the work. RH, PS, and MD drafted the report. RH, RR, GMS, and VW undertook literature searches, and gathered and harmonised data. All members of the writing committee participated in the drafting of the original manuscript. All members of the coordinating centre participated in the data collection and analysis. The data management team harmonised all data. PS performed the statistical analysis. MD is the guarantor. The corresponding author affirms that all listed authors meet authorship criteria and that no others meeting the criteria have been omitted.

Ethical approval: All original studies operated under supervision of an appropriate human ethics committee. This meta-analysis is exempt from ethics approval because the study collected data from previous clinical studies in which informed consent was already obtained.

Data sharing: Requests for patient level data will be considered by the COME-CCT Consortium.

The lead author affirms that the manuscript is an honest, accurate, and transparent account of the study being reported; that no important aspects of the study have been omitted; and that any discrepancies from the study as planned (and, if relevant, registered) have been explained.
