# Supplementary material for: Diagnosis of obstructive coronary artery disease using computed tomography angiography in patients with stable chest pain depending on clinical probability and in clinically important subgroups: meta-analysis of individual patient data
Source: BMJ. 2019 Jun 12;365:l1945. doi: 10.1136/bmj.l1945 (PMC6561308; doi:10.1136/bmj.l1945)
Supplement: Supplementary file 2 — Web appendix 2: Supplementary materials [file haar046861.ww2.pdf]

Supplementary table 1 | Characteristics of studies included in this analysis.

Study characteristics and citation details					Study data contribution	
**Study no.**	**First author**	**Journal**	**Year**	**Title**	**Pts. in data set***	**Data set no.**
1	Leschka S	Eur Heart J	2005	Accuracy of MSCT coronary angiography with 64-slice technology: first experience	49	**1**
2	Alkadhi H	Heart	2010	Low-dose, 128-slice, dual-source CT coronary angiography: accuracy and radiation dose of the high-pitch and the step-and-shoot mode	99	**2**
3	Alkadhi H	Eur Heart J	2008	Dual-source computed tomography coronary angiography: influence of obesity, calcium load, and heart rate on diagnostic accuracy	150	**3**
4	Leschka S	AJR Am J Roentgenol	2008	Effect of decrease in heart rate variability on the diagnostic accuracy of 64-MDCT coronary angiography	80	**4**
5	Andreini D	J Am Coll Cardiol	2007	Diagnostic accuracy of multidetector computed tomography coronary angiography in patients with dilated cardiomyopathy	170	**5**
6	Andreini D	Circ Cardiovasc Imaging	2009	Sixty-four-slice multidetector computed tomography: an accurate imaging modality for the evaluation of coronary arteries in dilated cardiomyopathy of unknown etiology	127	**6**
7	Bettencourt N	Circ Cardiovasc Imaging	2009	Multislice computed tomography in the exclusion of coronary artery disease in patients with presurgical valve disease	65	**7**
8	Dewey M	Ann Intern Med	2006	Noninvasive detection of coronary artery stenoses with multislice computed tomography or magnetic resonance imaging	129	**8**
9	Dewey M	Circulation	2009	Noninvasive coronary angiography by 320-row computed tomography with lower radiation exposure and maintained diagnostic accuracy: comparison of results with cardiac catheterization in a head-to-head pilot investigation	29	**9**
10	Chow BJ	Can J Cardiol	2007	Comparison of computed tomographic angiography versus rubidium-82 positron emission tomography for the detection of patients with anatomical coronary artery disease	26	**10**
11	Diederichsen AC	Scand Cardiovasc J	2009	Diagnostic value of cardiac 64-slice computed tomography: importance of coronary calcium	99	**11**

12	Diederichsen AC	Unpublished study #1			50	**12**
13	Garcia MJ	JAMA	2006	Accuracy of 16-row multidetector computed tomography for the assessment of coronary artery stenosis	230	**13**
14	Watkins MW	Am J Cardiol	2007	Detection of coronary artery stenosis using 40-channel computed tomography with multi-segment reconstruction	8	**14**
15	Kefer J	J Am Coll Cardiol	2005	Head-to-head comparison of three-dimensional navigator-gated magnetic resonance imaging and 16-slice computed tomography to detect coronary artery stenosis in patients	41	**15**
16	Pouleur AC	Circ Cardiovasc Imaging	2008	Direct comparison of whole-heart navigator-gated magnetic resonance coronary angiography and 40- and 64-slice multidetector row computed tomography to detect the coronary artery stenosis in patients scheduled for conventional coronary angiography	75	**16**
17	Pouleur AC	European Radiology	2007	Usefulness of 40-slice multidetector row computed tomography to detect coronary disease in patients prior to cardiac valve surgery	15	**17**
18	Ghostine S	J Am Coll Cardiol	2006	Non-invasive detection of coronary artery disease in patients with left bundle branch block using 64-slice computed tomography	32	**18**
19	Halon DA	Cardiology	2007	Uses and limitations of 40 slice multi-detector row spiral computed tomography for diagnosing coronary lesions in unselected patients referred for routine invasive coronary angiography	42	**19**
20	Halvorsen BA	Tidsskr Nor Laegeforen	2008	Angiography with 64-channel CT upon suspicion of stable coronary disease	83	**20**
21	Hamdan A	JACC Cardiovasc Imaging	2011	A prospective study for comparison of MR and CT imaging for detection of coronary artery stenosis	88	**21**
22	Hausleiter J	Eur Heart J	2007	Non-invasive coronary computed tomographic angiography for patients with suspected coronary artery disease: the Coronary Angiography by Computed Tomography with the Use of a Submillimeter resolution (CACTUS) trial	243	**22**
23	Jenkins SM	QJM	2011	Limited clinical utility of CT coronary angiography in a district hospital setting	99	**23**

24	Langer C	J Comput Assist Tomogr	2009	Noninvasive coronary angiography focusing on calcification: multislice computed tomography compared with magnetic resonance imaging	68	**24**
25	Martuscelli E	Eur Heart J	2004	Accuracy of thin-slice computed tomography in the detection of coronary stenoses	64	**25**
26	Meijboom WB	Am J Cardiol	2007	Comparison of diagnostic accuracy of 64-slice computed tomography coronary angiography in women versus men with angina pectoris	178	**26 and 27**
27	Meijboom WB	Heart	2007	64-Slice CT coronary angiography in patients with non-ST elevation acute coronary syndrome		
28	Meijboom WB	J Am Coll Cardiol	2006	Pre-operative computed tomography coronary angiography to detect significant coronary artery disease in patients referred for cardiac valve surgery		
29	Mollet NR	Circulation	2005	High-resolution spiral computed tomography coronary angiography in patients referred for diagnostic conventional coronary angiography		
30	Pugliese F	Eur Radiol	2006	Diagnostic accuracy of non-invasive 64-slice CT coronary angiography in patients with stable angina pectoris		
31	Meijboom WB	J Am Coll Cardiol	2008	Diagnostic accuracy of 64-slice computed tomography coronary angiography: a prospective, multicenter, multivendor study	108	**28**
32	Mendoza-Rodriguez V	The Internet Journal of Cardiology	2009	Ischemic Heart Disease Diagnosed by 64 Slice Computed Tomography Coronary Angiography	81	**29**
33	Bonmassari R	J Cardiovasc Med (Hagerstown)	2006	Noninvasive detection of coronary artery stenosis with 16-slice spiral computed tomography in a population at low to moderate risk for coronary artery disease	33	**30**
34	Nikolaou K	AJR Am J Roentgenol	2006	Accuracy of 64-MDCT in the diagnosis of ischemic heart disease	60	**31**
35	Nikolaou K	AJR Am J Roentgenol	2006	Clinical value of MDCT in the diagnosis of coronary artery disease in patients with a low pretest likelihood of significant disease	33	**32**
36	Ovrehus KA	Am J Cardiol	2010	Comparison of usefulness of exercise testing versus coronary computed tomographic angiography for evaluation of patients suspected of having coronary artery disease	100	**33**

37	Ovrehus KA	JCCT	2010	Coronary computed tomographic angiography in patients suspected of coronary artery disease: impact of observer experience on diagnostic performance and interobserver reproducibility	110	**34**
38	Pontone G	Clin Radiol	2007	Accuracy of multidetector spiral computed tomography in detecting significant coronary stenosis in patient populations with differing pre-test probabilities of disease	116	**35**
39	Pontone G	Coron Artery Dis	2007	Diagnostic work-up of unselected patients with suspected coronary artery disease: complementary role of multidetector computed tomography, symptoms and electrocardiogram stress test	144	**36**
40	Pontone G	J Am Coll Cardiol	2009	Diagnostic accuracy of coronary computed tomography angiography: a comparison between prospective and retrospective electrocardiogram triggering	96	**37**
41	Rixe J	Circ J	2009	Detection of relevant coronary artery disease using dual-source computed tomography in a high probability patient series: comparison with invasive angiography	76	**38**
42	Sato A	J Nucl Cardiol	2010	Incremental value of combining 64-slice computed tomography angiography with stress nuclear myocardial perfusion imaging to improve noninvasive detection of coronary artery disease	107	**39**
43	Herzog C	Radiology	2007	Does two-segment image reconstruction at 64-section CT coronary angiography improve image quality and diagnostic accuracy?	98	**40**
44	Herzog C	Radiology	2007	Significant coronary artery stenosis: comparison on per-patient and per-vessel or per-segment basis at 64-section CT angiography		
45	Arnoldi E	Radiologe	2010	Coronary CT angiography using prospective ECG triggering: high diagnostic accuracy with low radiation dose	20	**41**
46	Shabestari AA	Am J Cardiol	2007	Diagnostic performance of 64-channel multislice computed tomography in assessment of significant coronary artery disease in symptomatic subjects	113	**42**
47	Ugolini P	Can J Cardiol	2009	Evaluation of coronary atheroma by 64-slice multidetector computed tomography: Comparison with intravascular ultrasound and angiography	13	**43**
48	Ugolini P	Unpublished study #2			3	**44**

49	Yang L	AJR Am J Roentgenol	2009	64-MDCT coronary angiography of patients with atrial fibrillation: influence of heart rate on image quality and efficacy in evaluation of coronary artery disease	60	**45**
50	Xu L	Eur Radiol	2011	Diagnostic performance of 320-detector CT coronary angiography in patients with atrial fibrillation: preliminary results	37	**46**
51	Sun K	Chinese Medical Sciences Journal	2012	Feasibility and diagnostic accuracy for assessment of coronary artery stenosis of prospectively electrocardiogram-gated high-pitch spiral acquisition mode dual-source ct coronary angiography in patients with relatively higher heart rates: In comparison with catheter coronary angiography	37	**47**
52	Jakamy R	Arch Cardiovasc Dis	2012	Accuracy of multislice computed tomography in the preoperative assessment of coronary disease in patients scheduled for heart valve surgery	32	**48**
53	Kajander	Circulation	2010	Cardiac positron emission tomography/computed tomography imaging accurately detects anatomically and functionally significant coronary artery disease	99	**49**
54	Pontone G	Am Heart J	2011	Feasibility and accuracy of a comprehensive multidetector computed tomography acquisition for patients referred for balloon-expandable transcatheter aortic valve implantation	39	**50**
55	Herzog BA	Eur Heart J	2008	Accuracy of low-dose computed tomography coronary angiography using prospective electrocardiogram-triggering: first clinical experience	22	**51**
56	Herzog BA	Heart	2009	First head-to-head comparison of effective radiation dose from low-dose 64-slice CT with prospective ECG-triggering versus invasive coronary angiography	36	**52**
57	Husmann L	Acad Radiol	2010	Usefulness of additional coronary calcium scoring in low-dose CT coronary angiography with prospective ECG-triggering impact on total effective radiation dose and diagnostic accuracy	61	**53**
58	Andreini D	Cardiovasc Diabetol	2010	Comparison of the diagnostic performance of 64-slice computed tomography coronary angiography in diabetic and non-diabetic patients with suspected coronary artery disease	210	**54**

59	Chen CC	Int J Cardiovasc Imaging	2011	The effect of calcium score on the diagnostic accuracy of coronary computed tomography angiography	75	**55**
60	Laissy JP	Heart	2007	Comprehensive evaluation of preoperative patients with aortic valve stenosis: usefulness of cardiac multidetector computed tomography	13	**56**
61	Scheffel H	Eur Radiol	2006	Accuracy of dual-source CT coronary angiography: First experience in a high pre-test probability population without heart rate control	24	**57**
62	Leschka S	Heart	2008	Combining dual-source computed tomography coronary angiography and calcium scoring: added value for the assessment of coronary artery disease	67	**58**
63	Gueret P	Am J Cardiol	2013	Diagnostic Performance of Computed Tomography Coronary Angiography (from the Prospective National Multicenter Multivendor EVASCAN Study)	574	**59**
64	Rochitte CE	Eur Heart J	2014	Computed tomography angiography and perfusion to assess coronary artery stenosis causing perfusion defects by single photon emission computed tomography: the CORE320 study	265	**60**
65	Ghostine S	Eur Heart J	2008	Non-invasive diagnosis of ischaemic heart failure using 64-slice computed tomography	31	**61**

Full study citations can be found in Supplementary table 18. Pts. = Number of patients *=number of patients in the data set for main analysis. Number in the whole COME-CCT data set may be higher. The data set number will be used in supplementary 5 and 6 for identification.

Supplementary table 2 | QUADAS-2 analysis

	Studies		Risk of Bias				Applicability Concerns			Risk of Bias	Applicability Concerns
#	First author	Journal, Year	Patient Selection	Index Test	Reference Standard	Flow and Timing	Patient Selection	Index Test	Reference Standard	**Total**	**Total**
1	Alkadhi H	Eur Heart J 2008	Low	Low	Low	Low	Low	Low	Low	**Low**	**Low**
2	Alkadhi H	Heart 2010	Low	Low	Low	Low	Low	Low	Low	**Low**	**Low**
3	Andreini D	Cardiovasc Diabetol 2010	Low	Low	Low	Low	Low	Low	Low	**Low**	**Low**
4	Andreini D	Circ Cardiovasc Imaging 2009	Low	Low	Low	Low	Low	Low	Low	**Low**	**Low**
5	Andreini D	J Am Coll Cardiol 2007	Low	Low	Low	Low	Low	Low	Low	**Low**	**Low**
6	Arnoldi E	Radiologe 2010	Low	Unclear	Low	Unclear	Low	Low	Low	**Unclear**	**Low**
7	Bettencourt N	Circ Cardiovasc Imaging 2009	Low	Low	Low	Low	Low	Low	Low	**Low**	**Low**
8	Bonmassari R	J Cardiovasc Med (Hagerstown) 2006	Low	Unclear	Unclear	Unclear	Low	Low	Low	**Unclear**	**Low**
9	Chen CC	Int J Cardiovasc Imaging 2011	Low	Low	Unclear	High	Low	Low	Low	**High**	**Low**
10	Chow BJ	Can J Cardiol 2007	Unclear	Unclear	Unclear	Low	Low	Low	Low	**Unclear**	**Low**
11	Dewey M	Ann Intern Med 2006	Low	Low	Low	Low	Low	Low	Low	**Low**	**Low**
12	Dewey M	Circulation 2009	Low	Low	Low	Low	Low	Low	Low	**Low**	**Low**
13	Diederichsen AC	Scand Cardiovasc J 2009	Low	Low	Low	Low	Low	Low	Low	**Low**	**Low**
14	Garcia MJ	JAMA 2006	High	Low	Unclear	Low	Low	Low	Low	**High**	**Low**
15	Ghostine S	J Am Coll Cardiol 2006	Low	Low	Low	Low	Low	Low	Low	**Low**	**Low**

16	Ghostine S	Eur Heart 2008	Low	Low	Low	Low	Low	Low	Low	**Low**	**Low**
17	Gueret P	Am J Cardiol 2013	High	Low	Low	Low	Low	Low	Low	**High**	**Low**
18	Halon DA	Cardiology 2007	High	Low	Low	High	Low	Low	Low	**High**	**Low**
19	Halvorsen BA	Tidsskr Nor Laegeforen 2008	X	X	X	X	X	X	X	X	X
20	Hamdan A	JACC Cardiovasc Imaging 2011	High	Low	Low	Low	Low	Low	Low	**High**	**Low**
21	Hausleiter J	Eur Heart J 2007	Low	Low	Low	Low	Low	Low	Low	**Low**	**Low**
22	Herzog BA	Eur Heart J 2008	Low	Unclear	Low	Low	Low	Low	Low	**Low**	**Low**
23	Herzog BA	Heart 2009	Low	Unclear	Low	Low	Low	Low	Low	**Low**	**Low**
24	Herzog C	Radiology 2007*	Low	Low	Low	Unclear	Low	Low	Low	**Unclear**	**Low**
25	Herzog C	Radiology 2007**	Low	Unclear	Low	Low	Low	Low	Low	**Unclear**	**Low**
26	Husmann L	Acad Radiol 2010	High	Unclear	Low	Unclear	Low	Low	Low	**High**	**Low**
27	Jakamy R	Arch Cardiovasc Dis 2012	Low	Unclear	Low	High	Low	Low	Low	**High**	**Low**
28	Jenkins SM	QJM 2012	Low	Low	Low	High	Low	Low	Low	**Low**	**Low**
29	Kajander	Circulation 2010	Low	Low	Low	High	Low	Low	Low	**High**	**Low**
30	Kefer J	J Am Coll Cardiol 2005	Low	Low	Low	High	Low	Low	Low	**Low**	**Low**
31	Laissy JP	Heart 2007	Low	Unclear	Unclear	Low	Low	Low	Low	**Unclear**	**Low**
32	Langer C	J Comput Assist Tomogr 2007	Low	Low	Unclear	Low	Low	Low	Low	**Unclear**	**Low**
33	Leschka S	Eur Heart 2005	High	Low	Low	Low	Low	Low	Low	**High**	**Low**
34	Leschka S	Heart 2008	Low	Low	Low	Low	Low	Low	Low	**Low**	**Low**
35	Leschka S	AJR Am J Roentgenol 2008	High	Low	Low	Low	Low	Low	Low	**High**	**Low**
36	Martuscelli E	Eur Heart J 2004	Low	Low	Unclear	High	Low	Low	Low	**High**	**Low**
37	Meijboom WB	J Am Coll Cardiol 2008	Low	Low	Low	Low	Low	Low	Low	**Low**	**Low**

38	Meijboom WB	J Am Coll Cardiol 2006	Low	Low	Low	Low	Low	Low	Low	**Low**	**Low**
39	Meijboom WB	Heart 2007	Low	Low	Low	Low	Low	Low	Low	**Low**	**Low**
40	Meijboom WB	Am J Cardiol 2007	Low	Low	Low	Low	Low	Low	Low	**Low**	**Low**
41	Mendoza-Rodriguez V	The Internet Journal of Cardiology 2007	Low	Unclear	Low	Unclear	Low	Low	Low	**Unclear**	**Low**
42	Mollet NR	Circulation 2005	Low	Low	Low	Low	Low	Low	Low	**Low**	**Low**
43	Nikolaou K	AJR Am J Roentgenol+	High	Low	Low	High	Low	Low	Low	**High**	**Low**
44	Nikolaou K	AJR Am J Roentgenol++	Low	Low	Low	Low	Low	Low	Low	**Low**	**Low**
45	Ovrehus KA	Am J Cardiol 2010	Low	Low	Low	Low	Low	Low	Low	**Low**	**Low**
46	Ovrehus KA	J Cardiovasc Comput Tomogr 2010	Low	Low	Low	Low	Low	Low	Low	**Low**	**Low**
47	Pontone G	Coron Art Dis 2007	High	Unclear	Low	Unclear	Low	Low	Low	**Unclear**	**Low**
48	Pontone G	Am Heart J 2011	High	Unclear	Unclear	Unclear	Low	Low	Low	**High**	**Low**
49	Pontone G	J Am Coll Cardiol 2009	High	Low	Low	Low	Low	Low	Low	**High**	**Low**
50	Pontone G	Clin Radiol 2007	Low	Unclear	Low	High	Low	Low	Low	**High**	**Low**
51	Pouleur AC	Eur Radiol 2007	Low	Low	Low	Low	Low	Low	Low	**Low**	**Low**
52	Pouleur AC	Circ Cardiovasc Imaging 2008	Low	Low	Low	Low	Low	Low	Low	**Low**	**Low**
53	Pugliese F	Eur Radiol 2006	Low	Low	Low	Low	Low	Low	Low	**Low**	**Low**
54	Rixe J	Circ J 2009	Low	Unclear	Low	Low	Low	Low	Low	**Unclear**	**Low**
55	Rochitte CE	Eur Heart J 2014	High	Low	Low	Low	Low	Low	Low	**High**	**Low**
56	Sato A	J Nucl Cardiol 2010	Low	Low	Unclear	High	Low	Low	Low	**High**	**Low**
57	Scheffel H	Eur Radiol 2006	Low	Unclear	Low	High	Low	Low	Low	**High**	**Low**
58	Shabestari AA	Am J Cardiol 2007	High	Low	Unclear	High	Low	Low	Low	**High**	**Low**
59	Sun K	Chin Med Sci J 2012	Low	Low	Low	Unclear	Low	Low	Low	**Unclear**	**Low**
60	Ugolini P	Can J Cardiol 2009	Low	Low	Low	Low	Low	Low	Low	**Low**	**Low**

61	Watkins MW	Am J Cardiol 2007	High	Low	Low	Unclear	Low	Low	Low	**High**	**Low**
62	Xu L	Eur Radiol 2011	Low	Low	Low	Low	Low	Low	Low	**Low**	**Low**
63	Yang L	AJR Am J Roentgenol 2009	Low	Low	Low	Low	Low	Low	Low	**Low**	**Low**
64	Unpublished data	Diederichsen AC	X	X	X	X	X	X	X	X	X
65	Unpublished data	Ugolini P	X	X	X	X	X	X	X	X	X

* refers to study 24 in supplementary table 1. ** refers to study 25 in supplementary table 1. + refers to study 43 in supplementary table 1. ++ refers to study 44 in supplementary table 1. X = QUADAS assessment not possible because of study language (Norwegian, #19) or unpublished data (#64 and #65).

Supplementary table 3 | QUADAS-2 risk of bias summary

Risk of bias	Patient selection	Index test	Reference standard	Flow and timing	Total
**Low**	47	47	52	41	31
**High**	14	0	0	12	20
**Unclear**	1	15	10	9	11

Supplementary table 4 | QUADAS-2 applicability concerns summary

Applicability concerns	Patient selection	Index test	Reference standard	Total
**Low**	62	62	62	62
**High**	0	0	0	0
**Unclear**	0	0	0	0

Supplementary table 5 | Participant characteristics for each data set. Figures are numbers (percentages) unless stated otherwise.

Data set*	Patients	Age mean (SD)	Male sex	Chest pain symptoms				Risk factor distribution					Patients with CAD
				Typical AP	Atypical AP	Non-anginal CD	Other CD	Arterial HTN	DM	HLD	Active Smokers	Former Smokers	
**1**	49	56 (13)	35 (71.4)	18 (36.7)	22 (44.9)	9 (18.4)	0 (0.0)	26 (53.1)	5 (10.2)	21 (42.9)	17 (34.7)	3 (6.1)	30 (61.2)
**2**	99	63 (8)	73 (73.7)	17 (17.2)	37 (37.4)	45 (45.5)	0 (0.0)	46 (46.5)	23 (23.2)	35 (35.4)	38 (38.4)	31 (31.3)	35 (35.4)
**3**	150	64 (12)	103 (68.7)	32 (21.3)	91 (60.7)	27 (18.0)	0 (0.0)	75 (50.0)	29 (19.3)	57 (38.0)	62 (41.3)	7 (4.7)	59 (39.3)
**4**	80	61 (11)	46 (57.5)	12 (15.0)	32 (40.0)	27 (33.8)	9 (11.3)	41 (51.3)	12 (15.0)	40 (50.0)	32 (40.0)	7 (8.8)	39 (48.8)
**5**	170	54 (8)	121 (71.2)	0 (0.0)	20 (11.8)	19 (11.2)	131 (77.1)	37 (21.8)	1 (0.6)	45 (26.5)	22 (12.9)	25 (14.7)	84 (49.4)
**6**	127	56 (7)	79 (62.2)	0 (0.0)	5 (3.9)	3 (2.4)	119 (93.7)	49 (38.6)	14 (11.0)	41 (32.3)	17 (13.4)	0 (0.0)	46 (36.2)
**7**	65	70 (8)	38 (58.5)	62 (95.4)	0 (0.0)	0 (0.0)	3 (4.6)	45 (69.2)	12 (18.5)	33 (50.8)	5 (7.7)	8 (12.3)	22 (33.8)
**8**	129	63 (9)	34 (26.4)	61 (47.3)	32 (24.8)	13 (10.1)	23 (17.8)	93 (72.1)	21 (16.3)	66 (51.2)	30 (23.3)	59 (45.7)	67 (51.9)
**9**	29	60 (10)	9 (31.0)	7 (24.1)	10 (34.5)	2 (6.9)	10 (34.5)	26 (89.7)	5 (17.2)	17 (58.6)	4 (13.8)	14 (48.3)	11 (37.9)
**10**	26	56 (9)	18 (69.2)	17 (65.4)	4 (15.4)	1 (3.8)	4 (15.4)	15 (57.7)	4 (15.4)	19 (73.1)	4 (15.4)	16 (61.5)	19 (73.1)
**11**	99	62 (11)	53 (53.5)	77 (77.8)	0 (0.0)	0 (0.0)	22 (22.2)	49 (49.5)	9 (9.1)	43 (43.4)	25 (25.3)	32 (32.3)	31 (31.3)
**12**	50	62 (10)	26 (52.0)	13 (26.0)	19 (38.0)	18 (36.0)	0 (0.0)	30 (60.0)	3 (6.0)	18 (36.0)	13 (26.0)	24 (48.0)	20 (40.0)
**13**	230	60 (9)	156 (67.8)	134 (58.3)	59 (25.7)	37 (16.1)	0 (0.0)	102 (44.3)	14 (6.1)	98 (42.6)	47 (20.4)	100 (43.5)	72 (31.3)
**14**	8	61 (10)	6 (75.0)	5 (62.5)	3 (37.5)	0 (0.0)	0 (0.0)	6 (75.0)	0 (0.0)	7 (87.5)	0 (0.0)	0 (0.0)	3 (37.5)
**15**	41	64 (13)	32 (78.0)	21 (51.2)	4 (9.8)	5 (12.2)	11 (26.8)	23 (56.1)	5 (12.2)	20 (48.8)	15 (36.6)	4 (9.8)	24 (58.5)
**16**	75	60 (13)	56 (74.7)	12 (16.0)	4 (5.3)	5 (6.7)	54 (72.0)	40 (53.3)	13 (17.3)	45 (60.0)	23 (30.7)	10 (13.3)	17 (22.7)
**17**	15	62 (12)	9 (60.0)	3 (20.0)	0 (0.0)	0 (0.0)	12 (80.0)	8 (53.3)	0 (0.0)	4 (26.7)	4 (26.7)	0 (0.0)	5 (33.3)
**18**	32	70 (13)	15 (46.9)	20 (62.5)	12 (37.5)	0 (0.0)	0 (0.0)	23 (71.9)	10 (31.3)	19 (59.4)	13 (40.6)	0 (0.0)	15 (46.9)
**19**	42	56 (12)	35 (83.3)	0 (0.0)	0 (0.0)	0 (0.0)	42 (100.0)	18 (42.9)	8 (19.0)	0 (0.0)	11 (26.2)	9 (21.4)	22 (52.4)
**20**	83	61 (9)	46 (55.4)	46 (55.4)	35 (42.2)	0 (0.0)	2 (2.4)	42 (50.6)	12 (14.5)	53 (63.9)	15 (18.1)	34 (41.0)	39 (47.0)
**21**	88	64 (9)	56 (63.6)	25 (28.4)	28 (31.8)	17 (19.3)	18 (20.5)	62 (70.5)	20 (22.7)	53 (60.2)	16 (18.2)	40 (45.5)	44 (50.0)
**22**	243	62 (10)	158 (65.0)	38 (15.6)	136 (56.0)	0 (0.0)	69 (28.4)	0 (0.0)	0 (0.0)	152 (62.6)	38 (15.6)	46 (18.9)	101 (41.6)
**23**	99	58 (11)	55 (55.6)	55 (55.6)	0 (0.0)	38 (38.4)	6 (6.1)	53 (53.5)	11 (11.1)	87 (87.9)	24 (24.2)	32 (32.3)	38 (38.4)
**24**	68	64 (11)	38 (55.9)	0 (0.0)	68 (100.0)	0 (0.0)	0 (0.0)	57 (83.8)	11 (16.2)	51 (75.0)	7 (10.3)	22 (32.4)	26 (38.2)

**25**	64	59 (7)	59 (92.2)	33 (51.6)	16 (25.0)	2 (3.1)	13 (20.3)	61 (95.3)	37 (57.8)	52 (81.3)	24 (37.5)	24 (37.5)	43 (67.2)
**26**	38	60 (12)	33 (86.8)	18 (47.4)	11 (28.9)	9 (23.7)	0 (0.0)	20 (52.6)	7 (18.4)	21 (55.3)	10 (26.3)	0 (0.0)	21 (55.3)
**27**	140	60 (11)	95 (67.9)	58 (41.4)	35 (25.0)	47 (33.6)	0 (0.0)	74 (52.9)	15 (10.7)	67 (47.9)	37 (26.4)	0 (0.0)	72 (51.4)
**28**	108	61 (6)	75 (69.4)	61 (56.5)	17 (15.7)	10 (9.3)	20 (18.5)	65 (60.2)	24 (22.2)	67 (62.0)	28 (25.9)	0 (0.0)	69 (63.9)
**29**	81	56 (8)	61 (75.3)	25 (30.9)	56 (69.1)	0 (0.0)	0 (0.0)	52 (64.2)	20 (24.7)	41 (50.6)	39 (48.1)	0 (0.0)	18 (22.2)
**30**	33	69 (8)	23 (69.7)	0 (0.0)	0 (0.0)	6 (18.2)	27 (81.8)	16 (48.5)	8 (24.2)	17 (51.5)	7 (21.2)	11 (33.3)	14 (42.4)
**31**	60	60 (10)	33 (55.0)	3 (5.0)	22 (36.7)	35 (58.3)	0 (0.0)	NA	NA	NA	NA	NA	5 (8.3)
**32**	33	64 (11)	24 (72.7)	32 (97.0)	1 (3.0)	0 (0.0)	0 (0.0)	NA	NA	NA	NA	NA	21 (63.6)
**33**	100	61 (9)	50 (50.0)	31 (31.0)	28 (28.0)	41 (41.0)	0 (0.0)	50 (50.0)	3 (3.0)	69 (69.0)	14 (14.0)	38 (38.0)	29 (29.0)
**34**	110	61 (9)	72 (65.5)	64 (58.2)	46 (41.8)	0 (0.0)	0 (0.0)	70 (63.6)	15 (13.6)	82 (74.5)	24 (21.8)	45 (40.9)	44 (40.0)
**35**	116	63 (10)	86 (74.1)	10 (8.6)	39 (33.6)	67 (57.8)	0 (0.0)	76 (65.5)	14 (12.1)	71 (61.2)	25 (21.6)	27 (23.3)	63 (54.3)
**36**	144	62 (10)	109 (75.7)	58 (40.3)	86 (59.7)	0 (0.0)	0 (0.0)	88 (61.1)	21 (14.6)	105 (72.9)	49 (34.0)	47 (32.6)	95 (66.0)
**37**	96	65 (9)	81 (84.4)	24 (25.0)	37 (38.5)	4 (4.2)	31 (32.3)	64 (66.7)	17 (17.7)	54 (56.3)	27 (28.1)	40 (41.7)	90 (93.8)
**38**	76	63 (10)	47 (61.8)	22 (28.9)	35 (46.1)	12 (15.8)	7 (9.2)	64 (84.2)	25 (32.9)	51 (67.1)	9 (11.8)	14 (18.4)	40 (52.6)
**39**	107	67 (10)	69 (64.5)	69 (64.5)	21 (19.6)	10 (9.3)	7 (6.5)	73 (68.2)	49 (45.8)	65 (60.7)	44 (41.1)	21 (19.6)	59 (55.1)
**40**	98	60 (10)	49 (50.0)	75 (76.5)	20 (20.4)	0 (0.0)	3 (3.1)	86 (87.8)	27 (27.6)	0 (0.0)	24 (24.5)	24 (24.5)	45 (45.9)
**41**	20	60 (12)	15 (75.0)	20 (100.0)	0 (0.0)	0 (0.0)	0 (0.0)	0 (0.0)	0 (0.0)	0 (0.0)	0 (0.0)	0 (0.0)	11 (55.0)
**42**	113	64 (10)	81 (71.7)	1 (0.9)	2 (1.8)	80 (70.8)	30 (26.5)	87 (77.0)	48 (42.5)	63 (55.8)	49 (43.4)	9 (8.0)	90 (79.6)
**43**	13	62 (5)	8 (61.5)	7 (53.8)	4 (30.8)	1 (7.7)	1 (7.7)	4 (30.8)	0 (0.0)	12 (92.3)	2 (15.4)	6 (46.2)	5 (38.5)
**44**	3	54 (16)	2 (66.7)	1 (33.3)	1 (33.3)	1 (33.3)	0 (0.0)	0 (0.0)	0 (0.0)	0 (0.0)	0 (0.0)	2 (66.7)	0 (0.0)
**45**	60	58 (7)	23 (38.3)	2 (3.3)	6 (10.0)	14 (23.3)	38 (63.3)	4 (6.7)	1 (1.7)	6 (10.0)	10 (16.7)	1 (1.7)	8 (13.3)
**46**	37	60 (6)	16 (43.2)	6 (16.2)	3 (8.1)	6 (16.2)	22 (59.5)	12 (32.4)	6 (16.2)	19 (51.4)	3 (8.1)	10 (27.0)	10 (27.0)
**47**	37	59 (11)	25 (67.6)	5 (13.5)	3 (8.1)	24 (64.9)	5 (13.5)	6 (16.2)	4 (10.8)	11 (29.7)	15 (40.5)	16 (43.2)	27 (73.0)
**48**	32	65 (12)	21 (65.6)	1 (3.1)	0 (0.0)	0 (0.0)	31 (96.9)	19 (59.4)	4 (12.5)	14 (43.8)	5 (15.6)	13 (40.6)	6 (18.8)
**49**	99	64 (7)	56 (56.6)	53 (53.5)	39 (39.4)	7 (7.1)	0 (0.0)	39 (39.4)	13 (13.1)	65 (65.7)	16 (16.2)	9 (9.1)	43 (43.4)
**50**	39	80 (9)	10 (25.6)	3 (7.7)	0 (0.0)	5 (12.8)	31 (79.5)	26 (66.7)	2 (5.1)	13 (33.3)	7 (17.9)	3 (7.7)	11 (28.2)
**51**	22	58 (11)	13 (59.1)	6 (27.3)	10 (45.5)	2 (9.1)	4 (18.2)	10 (45.5)	3 (13.6)	7 (31.8)	10 (45.5)	0 (0.0)	12 (54.5)
**52**	36	62 (9)	24 (66.7)	7 (19.4)	19 (52.8)	9 (25.0)	1 (2.8)	21 (58.3)	3 (8.3)	19 (52.8)	18 (50.0)	0 (0.0)	20 (55.6)
**53**	61	61 (11)	37 (60.7)	35 (57.4)	11 (18.0)	12 (19.7)	3 (4.9)	36 (59.0)	4 (6.6)	28 (45.9)	28 (45.9)	0 (0.0)	33 (54.1)

**54**	210	61 (8)	184 (87.6)	38 (18.1)	45 (21.4)	34 (16.2)	93 (44.3)	137 (65.2)	105 (50.0)	104 (49.5)	60 (28.6)	0 (0.0)	180 (85.7)
**55**	75	61 (10)	61 (81.3)	31 (41.3)	31 (41.3)	6 (8.0)	7 (9.3)	48 (64.0)	15 (20.0)	23 (30.7)	14 (18.7)	14 (18.7)	50 (66.7)
**56**	13	69 (11)	7 (53.8)	3 (23.1)	1 (7.7)	1 (7.7)	8 (61.5)	6 (46.2)	2 (15.4)	4 (30.8)	2 (15.4)	0 (0.0)	2 (15.4)
**57**	24	63 (12)	18 (75.0)	15 (62.5)	2 (8.3)	7 (29.2)	0 (0.0)	17 (70.8)	13 (54.2)	14 (58.3)	19 (79.2)	0 (0.0)	9 (37.5)
**58**	67	61 (12)	44 (65.7)	34 (50.7)	15 (22.4)	7 (10.4)	11 (16.4)	39 (58.2)	16 (23.9)	16 (23.9)	17 (25.4)	0 (0.0)	29 (43.3)
**59**	574	60 (11)	387 (67.4)	320 (55.7)	189 (32.9)	65 (11.3)	0 (0.0)	296 (51.6)	136 (23.7)	180 (31.4)	157 (27.4)	0 (0.0)	274 (47.7)
**60**	265	62 (8)	156 (58.9)	92 (34.7)	118 (44.5)	6 (2.3)	49 (18.5)	NA	NA	NA	NA	NA	152 (57.4)
**61**	31	69 (10)	22 (71.0)	29 (93.5)	2 (6.5)	0 (0.0)	0 (0.0)	17 (54.8)	7 (22.6)	17 (54.8)	14 (45.2)	0 (0.0)	20 (64.5)

* data set numbers for studies are provided in supplementary Table 1.

Supplementary table 6 | Technical characteristics of imaging tests for each data set. Figures are numbers (percentages) unless stated otherwise.

Study ID	Patients	CT rows						CT gating	CTA showing CAD	Effective dose, mean (SD), mSv Effective dose, mean (SD), mSv	kV
		16	32	40	64	128	320				
**1**	49	0	49	0	0	0	0	retrospective	30	10.0 (1.8)	120
**2**	99	0	0	0	0	99	0	prospective	43	0.9 (0.2)	100
**3**	150	0	0	0	150	0	0	retrospective	78	8.0 (1.0)	120
**4**	80	0	80	0	0	0	0	retrospective	53	10.1 (1.9)	120
**5**	170	170	0	0	0	0	0	retrospective	106	14.5 (1.1)	120 to 140 according to the patient's body weight
**6**	127	0	0	0	127	0	0	both	66	19.8 (6.9)	120
**7**	65	0	65	0	0	0	0	retrospective	55	11.2 (3.1)	120
**8**	129	129	0	0	0	0	0	retrospective	91	11.9 (1.4)	120
**9**	29	0	0	0	0	0	29	prospective	12	6.3 (4.0)	120
**10**	26	26	0	0	0	0	0	retrospective	18	NA	120
**11**	99	0	0	0	99	0	0	retrospective	48	25.5 (6.5)	120
**12**	50	0	0	0	50	0	0	both	58	7.4 (6.0)	NA
**13**	230	230	0	0	0	0	0	retrospective	190	6.3 (2.5)	120 to 140 according to the patient's body weight
**14**	8	0	0	8	0	0	0	retrospective	3	10.5 (1.5)	120 to 140 according to the patient's body weight
**15**	41	41	0	0	0	0	0	retrospective	31	11.5 (2.1)	140
**16**	75	1	0	58	16	0	0	retrospective	30	13.6 (2.3)	120
**17**	15	0	0	15	0	0	0	retrospective	5	13.8 (2.1)	120
**18**	32	0	0	0	32	0	0	retrospective	17	7.2 (2.4)	120
**19**	42	0	0	42	0	0	0	retrospective	27	NA	120

**20**	83	0	0	0	83	0	0	retrospective	52	NA	120 to 135
**21**	88	0	88	0	0	0	0	retrospective	56	16.5 (3.8)	120
**22**	243	129	0	0	114	0	0	retrospective	136	7.3 (3.0)	Not explicitly stated*
**23**	99	0	0	99	0	0	0	retrospective	99	15.7 (3.0)	120 to 140 according to the patient's body weight
**24**	68	68	0	0	0	0	0	retrospective	65	7.7 (3.8)	120
**25**	64	64	0	0	0	0	0	retrospective	46	NA	120
**26**	38	0	38	0	0	0	0	retrospective	22	NA	120
**27**	140	0	140	0	0	0	0	retrospective	80	NA	120
**28**	108	0	0	0	0	0	0	retrospective	87	NA	120
**29**	81	0	81	0	0	0	0	retrospective	22	10.9 (1.8)	120
**30**	33	33	0	0	0	0	0	retrospective	36	NA	140
**31**	60	60	0	0	0	0	0	retrospective	39	14.3 (1.0)	120
**32**	33	33	0	0	0	0	0	retrospective	29	8.6 (1.5)	120
**33**	100	0	51	0	49	0	0	retrospective	47	NA	120
**34**	110	0	44	0	66	0	0	retrospective	61	14.5 (4.0)	120
**35**	116	116	0	0	0	0	0	retrospective	91	13.1 (2.3)	120
**36**	144	144	0	0	0	0	0	retrospective	117	13.2 (2.5)	120 to 140 according to the patient's body weight
**37**	96	0	0	0	96	0	0	both	91	14.1 (8.9)	120
**38**	76	0	0	0	76	0	0	retrospective	52	NA	120
**39**	107	0	0	0	107	0	0	retrospective	95	15.0 (0.0)	120
**40**	98	0	0	0	98	0	0	retrospective	38	NA	120
**41**	20	0	0	0	20	0	0	prospective	15	2.5 (1.1)	100 to 120 according to the patient's body weight
**42**	113	0	113	0	0	0	0	retrospective	94	14.1 (1.5)	120
**43**	13	0	0	0	13	0	0	retrospective	7	NA	120
**44**	3	0	0	0	3	0	0	retrospective	2	8.3 (4.4)	NA
**45**	60	0	0	0	60	0	0	retrospective	18	14.4 (4.2)	100 to 135

**46**	37	0	0	0	0	0	37	prospective	15	13.0 (4.7)	100 and 120
**47**	37	0	0	0	0	37	0	prospective	34	1.1 (0.1)	100
**48**	32	0	0	0	32	0	0	prospective	11	22.7 (14.4)	120
**49**	99	0	0	0	99	0	0	both	46	9.5 (5.2)	100 to 120, depending on patient size
**50**	39	0	0	0	39	0	0	retrospective	17	36.6 (13.9)	120
**51**	22	0	0	0	22	0	0	prospective	18	2.1 (0.8)	100 and 120, according to the patient's BMI
**52**	36	0	0	0	36	0	0	prospective	22	2.1 (0.7)	100 and 120, according to the patient's BMI
**53**	61	0	0	0	61	0	0	prospective	39	2.1 (0.7)	100 and 120, according to the patient's BMI
**54**	210	0	0	0	210	0	0	retrospective	192	14.6 (1.7)	120
**55**	75	0	0	0	75	0	0	retrospective	51	16.3 (1.8)	120
**56**	13	13	0	0	0	0	0	retrospective	3	NA	no information
**57**	24	0	0	0	0	24	0	retrospective	8	NA	120
**58**	67	0	0	0	0	67	0	retrospective	39	NA	120
**59**	574	0	0	0	574	0	0	prospective	608	18.3 (7.3)	100 to 140, according to the patient's body weight
**60**	79	0	0	0	0	0	79	retrospective	34	NA	120
**61**	31	0	0	0	31	0	0	retrospective	21	9.9 (5.0)	120

* original citations from the publication: "To decrease radiation exposure, we consequently used dose-saving algorithms including prospective ECG-gated tube current modulation and 100 kV acquisition protocols, whenever possible."

Supplementary Table 7 | Empiral data of female patients and their assignment to pretest probability categories

Female patients	Participants	Pretest probability categories									
	Overall	0 to <10%	10 to <20%	20 to < 30%	30 to <40%	40 to <50%	50 to <60%	60 to <70%	70 to <80%	80 to <90%	90 to 100%
**N**	1859	86	501	390	218	169	245	199	51	0	0
**TP**	533	14	114	97	69	52	90	68	29	-	-
**TN**	927	52	292	210	104	81	107	72	9	-	-
**FP**	147	6	35	40	21	11	17	12	5	-	-
**FN**	34	1	3	7	4	4	8	7	0	-	-
**NDX**	218	13	57	36	20	21	23	40	8	-	-
**NDX rate (%)**	11.7	15.1	11.4	9.2	9.2	12.4	9.4	20.1	15.7	-	-
**PPV (%)**	78.4	70.0	76.5	70.8	76.7	82.5	84.1	85.0	85.3	-	-
**NPV (%)**	96.5	98.1	99.0	96.8	96.3	95.3	93.0	91.1	100.0	-	-
**Sensitivity (%)**	94.0	93.3	97.4	93.3	94.5	92.9	91.8	90.7	100.0	-	-
**Specificity (%)**	86.3	89.7	89.3	84.0	83.2	88.0	86.3	85.7	64.3	-	-
**Diagnostic accuracy (%)**	89.0	90.4	91.4	86.7	87.4	89.9	88.7	88.1	88.4	-	-
**LR+**	6.87	9.02	9.10	5.83	5.63	7.77	6.70	6.35	2.80	-	-
**LR-**	0.07	0.07	0.03	0.08	0.07	0.08	0.09	0.11	0.00	-	-

Supplementary Table 8 | Empirical data of male patients and their assignment to pretest probability categories

Male patients	Participants	Pretest probability categories									
	Overall	0 to <10%	10 to <20%	20 to < 30%	30 to <40%	40 to <50%	50 to <60%	60 to <70%	70 to <80%	80 to <90%	90 to 100%
**N**	3473	0	29	211	509	576	507	391	484	698	68
**TP**	1718	-	6	79	203	269	220	188	288	420	45
**TN**	1104	-	21	102	230	206	187	122	94	134	8
**FP**	228	-	1	13	28	48	38	27	33	38	2
**FN**	87	-	0	3	14	7	9	15	17	21	1
**NDX**	336	-	1	14	34	46	53	39	52	85	12
**NDX rate (%)**	9,7	-	3.4	6.6	6.7	8.0	10.5	10.0	10.7	12.2	17.6
**PPV (%)**	88,3	-	85.7	85.9	87.9	84.9	85.3	87.4	89.7	91.7	95.7
**NPV (%)**	92,7	-	100.0	97.1	94.3	96.7	95.4	89.1	84.7	86.5	88.9
**Sensitivity (%)**	95,2	-	100.0	96.3	93.5	97.5	96.1	92.6	94.4	95.2	97.8
**Specificity (%)**	82,9	-	95.5	88.7	89.1	81.1	83.1	81.9	74.0	77.9	80.0
**Diagnostic accuracy (%)**	90,0	-	96.4	91.9	91.2	89.6	89.6	88.1	88.4	90.4	94.6
**LR+**	5,56	-	22.00	8.52	8.62	5.16	5.69	5.11	3.63	4.31	4.89
**LR-**	0,06	-	0.00	0.04	0.07	0.03	0.05	0.09	0.08	0.06	0.03

Supplementary table 9 | Predictors of non-diagnostic CTA results

**Random effects**		
Group	Variance	Standard deviation
Study_No (Intercept)	2.632	1.622

**Fixed effects**				
Group	Estimate	Standard error	Z value	P value
Intercept	-5.066	0.415	-12.242	<0.001
Age >75	-0.009	0.166	-0.055	0.9560
Male	-0.200	0.107	-1.877	0.605
Heart rate	0.028	0.005	5.990	<0.001

Supplementary Table 10 | Patients ≤50 years and their assignment to pretest probability categories

Patients ≤ 50 years	Participants	Pretest probability categories									
	Overall	0 to <10%	10 to <20%	20 to < 30%	30 to <40%	40 to <50%	50 to <60%	60 to <70%	70 to <80%	80 to <90%	90 to 100%
**N**	817	86	113	185	120	107	3	107	96	0	0
**TP**	255	14	19	69	26	39	0	40	48	-	-
**TN**	397	52	71	89	71	49	2	40	23	-	-
**FP**	51	6	5	12	6	7	1	7	7	-	-
**FN**	21	1	2	3	1	2	0	6	6	-	-
**NDX**	93	13	16	12	16	10	0	14	12	-	-
**NDX rate (%)**	11.4	15.1	14.2	6.5	13.3	9.3	0.0	13.1	12.5	-	-
**PPV (%)**	83.3	70.0	79.2	85.2	81.3	84.8	0.0	85.1	87.3	-	-
**NPV (%)**	95.0	98.1	97.3	96.7	98.6	96.1	100.0	87.0	79.3	-	-
**Sensitivity (%)**	92.4	93.3	90.5	95.8	96.3	95.1	n/a*	87.0	88.9	-	-
**Specificity (%)**	88.6	89.7	93.4	88.1	92.2	87.5	66.7	85.1	76.7	-	-
**Diagnostic accuracy (%)**	90.1	90.4	92.8	91.3	93.3	90.7	66.7	86.0	84.5	-	-
**LR+**	8.12	9.02	13.75	8.07	12.36	7.61	n/a*	5.84	3.81	-	-
**LR-**	0.09	0.07	0.10	0.05	0.04	0.06	n/a*	0.15	0.14	-	-

* n/a = not applicable (division by 0)

Supplementary Table 11 | Patients >50 to ≤65 years and their assignment to pretest probability categories

Patients >50 to ≤ 65 years	Participants	Pretest probability categories									
	Overall	0 to <10%	10 to <20%	20 to < 30%	30 to <40%	40 to <50%	50 to <60%	60 to <70%	70 to <80%	80 to <90%	90 to 100%
**N**	2619	0	344	242	432	425	538	0	317	321	0
**TP**	1100	-	83	50	186	171	215	-	201	194	-
**TN**	1039	-	201	140	182	172	223	-	54	67	-
**FP**	173	-	24	21	25	31	36	-	19	17	-
**FN**	48	-	1	3	14	7	11	-	8	4	-
**NDX**	259	-	35	28	25	44	53	-	35	39	-
**NDX rate (%)**	9.9	-	10.2	11.6	5.8	10.4	9.9	-	11.0	12.1	-
**PPV (%)**	86.4	-	77.6	70.4	88.2	84.7	85.7	-	91.4	91.9	-
**NPV (%)**	95.6	-	99.5	97.9	92.9	96.1	95.3	-	87.1	94.4	-
**Sensitivity (%)**	95.8	-	98.8	94.3	93.0	96.1	95.1	-	96.2	98.0	-
**Specificity (%)**	85.7	-	89.3	87.0	87.9	84.7	86.1	-	74.0	79.8	-
**Diagnostic accuracy (%)**	90.6	-	91.9	88.8	90.4	90.0	90.3	-	90.4	92.6	-
**LR+**	6.71	-	9.26	7.23	7.70	6.29	6.84	-	3.70	4.84	-
**LR-**	0.05	-	0.01	0.07	0.08	0.05	0.06	-	0.05	0.03	-

Supplementary Table 12 | Patients >65 to ≤ 75 years and their assignment to pretest probability categories

Patients >65 to ≤ 75 years	Participants	Pretest probability categories									
	Overall	0 to <10%	10 to <20%	20 to < 30%	30 to <40%	40 to <50%	50 to <60%	60 to <70%	70 to <80%	80 to <90%	90 to 100%
**N**	1434	0	73	116	133	180	160	434	0	338	0
**TP**	692	-	18	42	53	98	71	199	-	211	-
**TN**	451	-	41	48	57	56	56	138	-	55	-
**FP**	109	-	7	14	13	15	13	28	-	19	-
**FN**	38	-	0	4	2	2	3	13	-	14	-
**NDX**	144	-	7	8	8	9	17	56	-	39	-
**NDX rate (%)**	10.0	-	9.6	6.9	6.0	5.0	10.6	12.9	-	11.5	-
**PPV (%)**	86.4	-	72.0	75.0	80.3	86.7	84.5	87.7	-	91.7	-
**NPV (%)**	92.2	-	100.0	92.3	96.6	96.6	94.9	91.4	-	79.7	-
**Sensitivity (%)**	94.8	-	100.0	91.3	96.4	98.0	95.9	93.9	-	93.8	-
**Specificity (%)**	80.5	-	85.4	77.4	81.4	78.9	81.2	83.1	-	74.3	-
**Diagnostic accuracy (%)**	88.6	-	89.4	83.3	88.0	90.1	88.8	89.2	-	89.0	-
**LR+**	4.87	-	6.86	4.04	5.19	4.64	5.09	5.57	-	3.65	-
**LR-**	0.06	-	0.00	0.11	0.04	0.03	0.05	0.07	-	0.08	-

Supplementary Table 13 | Patients >75 years and their assignment to pretest probability categories

Patients >75 years	Participants	Pretest probability categories									
	Overall	0 to <10%	10 to <20%	20 to < 30%	30 to <40%	40 to <50%	50 to <60%	60 to <70%	70 to <80%	80 to <90%	90 to 100%
**N**	462	0	0	58	42	33	51	49	122	39	68
**TP**	204	-	-	15	7	13	24	17	68	15	45
**TN**	144	-	-	35	24	10	13	16	26	12	8
**FP**	42	-	-	6	5	6	5	4	12	2	2
**FN**	14	-	-	0	1	0	3	3	3	3	1
**NDX**	58	-	-	2	5	4	6	9	13	7	12
**NDX rate (%)**	12.6	-	-	3.4	11.9	12.1	11.8	18.4	10.7	17.9	17.6
**PPV (%)**	82.9	-	-	71.4	58.3	68.4	82.8	81.0	85.0	88.2	95.7
**NPV (%)**	91.1	-	-	100.0	96.0	100.0	81.3	84.2	89.7	80.0	88.9
**Sensitivity (%)**	93.6	-	-	100.0	87.5	100.0	88.9	85.0	95.8	83.3	97.8
**Specificity (%)**	77.4	-	-	85.4	82.8	62.5	72.2	80.0	68.4	85.7	80.0
**Diagnostic accuracy (%)**	86.1	-	-	89.3	83.8	79.3	82.2	82.5	86.2	84.4	94.6
**LR+**	4.14	-	-	6.83	5.08	2.67	3.20	4.25	3.03	5.83	4.89
**LR-**	0.08	-	-	0.00	0.15	0.00	0.15	0.19	0.06	0.19	0.03

Supplementary Table 14 | Patients with typical angina and their assignment to pretest probability categories

Typical angina	Participants	Pretest probability categories									
	**Overall**	**0 to <10%**	**10 to <20%**	**20 to < 30%**	**30 to <40%**	**40 to <50%**	**50 to <60%**	**60 to <70%**	**70 to <80%**	**80 to <90%**	**90 to 100%**
**N**	1967	0	0	4	43	137	247	306	464	698	68
**TP**	989	-	-	1	9	39	89	108	278	420	45
**TN**	547	-	-	3	23	72	109	112	86	134	8
**FP**	116	-	-	0	3	5	18	19	31	38	2
**FN**	62	-	-	0	1	4	8	13	14	21	1
**NDX**	253	-	-	0	7	17	23	54	55	85	12
**NDX rate (%)**	12.9	-	-	0.0	16.3	12.4	9.3	17.6	11.9	12.2	17.6
**PPV (%)**	89.5	-	-	100.0	75.0	88.6	83.2	85.0	90.0	91.7	95.7
**NPV (%)**	89.8	-	-	100.0	95.8	94.7	93.2	89.6	86.0	86.5	88.9
**Sensitivity (%)**	94.1	-	-	100.0	90.0	90.7	91.8	89.3	95.2	95.2	97.8
**Specificity (%)**	82.5	-	-	100.0	88.5	93.5	85.8	85.5	73.5	77.9	80.0
**Diagnostic accuracy (%)**	89.6	-	-	100.0	88.9	92.5	88.4	87.3	89.0	90.4	94.6
**LR+**	5.38	-	-	n/a*	7.80	13.97	6.47	6.15	3.59	4.31	4.89
**LR-**	0.07	-	-	0.00	0.11	0.10	0.10	0.13	0.07	0.06	0.03

* n/a = not applicable (division by 0)

Supplementary Table 15 | Patients with atypical angina and their assignment to pretest probability categories

Atypical angina	Participants	Pretest probability categories									
	**Overall**	**0 to <10%**	**10 to <20%**	**20 to < 30%**	**30 to <40%**	**40 to <50%**	**50 to <60%**	**60 to <70%**	**70 to <80%**	**80 to <90%**	**90 to 100%**
**N**	1592	1	138	269	235	280	339	260	70	0	0
**TP**	579	0	23	53	73	110	142	140	38	-	-
**TN**	691	1	86	150	120	108	136	73	17	-	-
**FP**	136	0	8	29	19	32	22	19	7	-	-
**FN**	27	0	2	5	2	4	4	7	3	-	-
**NDX**	159	0	19	32	21	26	35	21	5	-	-
**NDX rate (%)**	10.0	0.0	13.8	11.9	8.9	9.3	10.3	8.1	7.1	-	-
**PPV (%)**	81.0	n/a*	74.2	64.6	79.3	77.5	86.6	88.1	84.4	-	-
**NPV (%)**	96.2	100.0	97.7	96.8	98.4	96.4	97.1	91.3	85.0	-	-
**Sensitivity (%)**	95.5	n/a*	92.0	91.4	97.3	96.5	97.3	95.2	92.7	-	-
**Specificity (%)**	83.6	100.0	91.5	83.8	86.3	77.1	86.1	79.3	70.8	-	-
**Diagnostic accuracy (%)**	88.6	100.0	91.6	85.7	90.2	85.8	91.4	89.1	84.6	-	-
**LR+**	5.81	n/a*	10.81	5.64	7.12	4.22	6.99	4.61	3.18	-	-
**LR-**	0.05	n/a*	0.09	0.10	0.03	0.05	0.03	0.06	0.10	-	-

* n/a = not applicable (division by 0)

Supplementary Table 16 | Patients with non-anginal chest discomfort and their assignment to pretest probability categories

Non-anginal chest discomfort	Participants	Pretest probability categories									
	Overall	0 to <10%	10 to <20%	20 to < 30%	30 to <40%	40 to <50%	50 to <60%	60 to <70%	70 to <80%	80 to <90%	90 to 100%
**N**	796	38	162	157	188	158	80	12	1	0	0
**TP**	294	6	37	64	72	73	35	6	1	-	-
**TN**	347	19	87	71	83	58	27	2	0	-	-
**FP**	55	4	16	11	9	10	4	1	0	-	-
**FN**	17	1	1	2	9	1	3	0	0	-	-
**NDX**	83	8	21	9	15	16	11	3	0	-	-
**NDX rate (%)**	10.4	21.1	13.0	5.7	8.0	10.1	13.8	25.0	0.0	-	-
**PPV (%)**	84.2	60.0	69.8	85.3	88.9	88.0	89.7	85.7	100.0	-	-
**NPV (%)**	95.3	95.0	98.9	97.3	90.2	98.3	90.0	100.0	n/a*	-	-
**Sensitivity (%)**	94.5	85.7	97.4	97.0	88.9	98.6	92.1	100.0	100.0	-	-
**Specificity (%)**	86.3	82.6	84.5	86.6	90.2	85.3	87.1	66.7	n/a*	-	-
**Diagnostic accuracy (%)**	89.9	83.3	87.9	91.2	89.6	92.3	89.9	88.9	100.0	-	-
**LR+**	6.91	4.93	6.27	7.23	9.09	6.71	7.14	3.00	n/a*	-	-
**LR-**	0.06	0.17	0.03	0.03	0.12	0.02	0.09	0.00	n/a*	-	-

* n/a = not applicable (division by 0

Supplementary Table 17 | Patients with other chest discomfort and their assignment to pretest probability categories

Other chest discomfort	Participants	Pretest probability categories									
	Overall	0 to <10 %	10 to <20 %	20 to < 30%	30 to <40 %	40 to <50 %	50 to <60 %	60 to <70 %	70 to <80 %	80 to <90 %	90 to 100 %
**N**	977	47	230	171	261	170	86	12	0	0	0
**TP**	389	8	60	58	118	99	44	2	-	-	-
**TN**	446	32	140	88	108	49	22	7	-	-	-
**FP**	68	2	12	13	18	12	11	0	-	-	-
**FN**	15	0	0	3	6	2	2	2	-	-	-
**NDX**	59	5	18	9	11	8	7	1	-	-	-
**NDX rate (%)**	6,0	10,6	7,8	5,3	4,2	4,7	8,1	8,3	-	-	-
**PPV (%)**	85.1	80.0	83.3	81.7	86.8	89.2	80.0	100.0	-	-	-
**NPV (%)**	96.7	100.0	100.0	96.7	94.7	96.1	91.7	77.8	-	-	-
**Sensitivity (%)**	96.3	100.0	100.0	95.1	95.2	98.0	95.7	50.0	-	-	-
**Specificity (%)**	86.8	94.1	92.1	87.1	85.7	80.3	66.7	100.0	-	-	-
**Diagnostic accuracy (%)**	91.0	95.2	94.3	90.1	90.4	91.4	83.5	81.8	-	-	-
**LR+**	7.28	17.00	12.67	7.39	6.66	4.98	2.87	n/a*	-	-	-
**LR-**	0.04	0.00	0.00	0.06	0.06	0.02	0.07	0.50	-	-	-

* n/a = not applicable (division by 0)

Supplementary table19 | References of included studies in this analysis

1. Alkadhi H, Scheffel H, Desbiolles L, et al. Dual-source computed tomography coronary angiography: influence of obesity, calcium load, and heart rate on diagnostic accuracy. *European heart journal* 2008; **29**(6): 766-76.
2. Alkadhi H, Stolzmann P, Desbiolles L, et al. Low-dose, 128-slice, dual-source CT coronary angiography: accuracy and radiation dose of the high-pitch and the step-and-shoot mode. *Heart* 2010; **96**(12): 933-8.
3. Andreini D, Pontone G, Bartorelli AL, et al. Comparison of the diagnostic performance of 64-slice computed tomography coronary angiography in diabetic and non-diabetic patients with suspected coronary artery disease. *Cardiovascular diabetology* 2010; **9**: 80.
4. Andreini D, Pontone G, Bartorelli AL, et al. Sixty-four-slice multidetector computed tomography: an accurate imaging modality for the evaluation of coronary arteries in dilated cardiomyopathy of unknown etiology. *Circulation Cardiovascular imaging* 2009; **2**(3): 199-205.
5. Andreini D, Pontone G, Pepi M, et al. Diagnostic accuracy of multidetector computed tomography coronary angiography in patients with dilated cardiomyopathy. *Journal of the American*

College of Cardiology 2007; **49**(20): 2044-50.
6. Arnoldi E, Ramos-Duran I, Abro JA, et al. [Coronary CT angiography using prospective ECG triggering: high diagnostic accuracy with low radiation dose]. *Der Radiologe* 2010; **50**(6): 500-6.
7. Bettencourt N, Rocha J, Carvalho M, et al. Multislice computed tomography in the exclusion of coronary artery disease in patients with presurgical valve disease. *Circulation Cardiovascular imaging* 2009; **2**(4): 306-13.
8. Bonmassari R, Muraglia S, Centonze M, Coser D, Stoppa G, Disertori M. Noninvasive detection of coronary artery stenosis with 16-slice spiral computed tomography in a population at low to moderate risk for coronary artery disease. *Journal of cardiovascular medicine* 2006; **7**(11): 817-25.
9. Chen CC, Chen CC, Hsieh IC, et al. The effect of calcium score on the diagnostic accuracy of coronary computed tomography angiography. *The international journal of cardiovascular imaging* 2011; **27 Suppl 1**: 37-42.
10. Chow BJ, Dennie C, Hoffmann U, et al. Comparison of computed tomographic angiography versus rubidium-82 positron emission tomography for the detection of patients with anatomical coronary artery disease. *The Canadian journal of cardiology* 2007; **23**(10): 801-7.
11. Dewey M, Teige F, Schnapauff D, et al. Noninvasive detection of coronary artery stenoses with multislice computed tomography or magnetic resonance imaging. *Annals of internal medicine* 2006; **145**(6): 407-15.
12. Dewey M, Zimmermann E, Deissenrieder F, et al. Noninvasive coronary angiography by 320-row computed tomography with lower radiation exposure and maintained diagnostic accuracy: comparison of results with cardiac catheterization in a head-to-head pilot investigation. *Circulation* 2009; **120**(10): 867-75.
13. Diederichsen AC, Petersen H, Jensen LO, et al. Diagnostic value of cardiac 64-slice computed tomography: importance of coronary calcium. *Scandinavian cardiovascular journal : SCJ* 2009; **43**(5): 337-44.
14. Garcia MJ, Lessick J, Hoffmann MH, Investigators CS. Accuracy of 16-row multidetector computed tomography for the assessment of coronary artery stenosis. *Jama* 2006; **296**(4): 403-11.
15. Ghostine S, Caussin C, Daoud B, et al. Non-invasive detection of coronary artery disease in patients with left bundle branch block using 64-slice computed tomography. *Journal of the American College of Cardiology* 2006; **48**(10): 1929-34.
16. Ghostine S, Caussin C, Habis M, et al. Non-invasive diagnosis of ischaemic heart failure using 64-slice computed tomography. *European heart journal* 2008; **29**(17): 2133-40.
17. Gueret P, Deux JF, Bonello L, et al. Diagnostic performance of computed tomography coronary angiography (from the Prospective National Multicenter Multivendor EVASCAN Study). *The American journal of cardiology* 2013; **111**(4): 471-8.
18. Halon DA, Gaspar T, Adawi S, et al. Uses and limitations of 40 slice multi-detector row spiral computed tomography for diagnosing coronary lesions in unselected patients referred for routine invasive coronary angiography. *Cardiology* 2007; **108**(3): 200-9.
19. Halvorsen BA, Rodevand O, Hagen G, Herud E, Mielczarek W, Molstad P. [Angiography with 64-channel CT upon suspicion of stable coronary disease]. *Tidsskrift for den Norske laegeforening : tidsskrift for praktisk medicin, ny raekke* 2008; **128**(19): 2172-6.
20. Hamdan A, Asbach P, Wellnhofer E, et al. A prospective study for comparison of MR and CT imaging for detection of coronary artery stenosis. *JACC Cardiovascular imaging* 2011; **4**(1): 50-61.
21. Hausleiter J, Meyer T, Hadamitzky M, et al. Non-invasive coronary computed tomographic angiography for patients with suspected coronary artery disease: the Coronary Angiography by Computed Tomography with the Use of a Submillimeter resolution (CACTUS) trial. *European heart journal* 2007; **28**(24): 3034-41.
22. Herzog BA, Husmann L, Burkhard N, et al. Accuracy of low-dose computed

tomography coronary angiography using prospective electrocardiogram-triggering: first clinical experience. *European heart journal* 2008; **29**(24): 3037-42.
23. Herzog BA, Wyss CA, Husmann L, et al. First head-to-head comparison of effective radiation dose from low-dose 64-slice CT with prospective ECG-triggering versus invasive coronary angiography. *Heart* 2009; **95**(20): 1656-61.
24. Herzog C, Nguyen SA, Savino G, et al. Does two-segment image reconstruction at 64-section CT coronary angiography improve image quality and diagnostic accuracy? *Radiology* 2007; **244**(1): 121-9.
25. Herzog C, Zwerner PL, Doll JR, et al. Significant coronary artery stenosis: comparison on per-patient and per-vessel or per-segment basis at 64-section CT angiography. *Radiology* 2007; **244**(1): 112-20.
26. Husmann L, Herzog BA, Burger IA, et al. Usefulness of additional coronary calcium scoring in low-dose CT coronary angiography with prospective ECG-triggering impact on total effective radiation dose and diagnostic accuracy. *Academic radiology* 2010; **17**(2): 201-6.
27. Jakamy R, Barthelemy O, Le Feuvre C, et al. Accuracy of multislice computed tomography in the preoperative assessment of coronary disease in patients scheduled for heart valve surgery. *Archives of cardiovascular diseases* 2012; **105**(8-9): 424-31.
28. Jenkins SM, Johnston N, Hawkins NM, et al. Limited clinical utility of CT coronary angiography in a district hospital setting. *QJM : monthly journal of the Association of Physicians* 2011; **104**(1): 49-57.
29. Kajander S, Joutsiniemi E, Saraste M, et al. Cardiac positron emission tomography/computed tomography imaging accurately detects anatomically and functionally significant coronary artery disease. *Circulation* 2010; **122**(6): 603-13.
30. Kefer J, Coche E, Legros G, et al. Head-to-head comparison of three-dimensional navigator-gated magnetic resonance imaging and 16-slice computed tomography to detect coronary artery stenosis in patients. *Journal of the American College of Cardiology* 2005; **46**(1): 92-100.
31. Laissy JP, Messika-Zeitoun D, Serfaty JM, et al. Comprehensive evaluation of preoperative patients with aortic valve stenosis: usefulness of cardiac multidetector computed tomography. *Heart* 2007; **93**(9): 1121-5.
32. Langer C, Peterschroder A, Franzke K, et al. Noninvasive coronary angiography focusing on calcification: multislice computed tomography compared with magnetic resonance imaging. *Journal of computer assisted tomography* 2009; **33**(2): 179-85.
33. Leschka S, Alkadhi H, Plass A, et al. Accuracy of MSCT coronary angiography with 64-slice technology: first experience. *European heart journal* 2005; **26**(15): 1482-7.
34. Leschka S, Scheffel H, Desbiolles L, et al. Combining dual-source computed tomography coronary angiography and calcium scoring: added value for the assessment of coronary artery disease. *Heart* 2008; **94**(9): 1154-61.
35. Leschka S, Scheffel H, Husmann L, et al. Effect of decrease in heart rate variability on the diagnostic accuracy of 64-MDCT coronary angiography. *AJR American journal of roentgenology* 2008; **190**(6): 1583-90.
36. Martuscelli E, Romagnoli A, D'Eliseo A, et al. Accuracy of thin-slice computed tomography in the detection of coronary stenoses. *European heart journal* 2004; **25**(12): 1043-8.
37. Meijboom WB, Meijs MF, Schuijf JD, et al. Diagnostic accuracy of 64-slice computed tomography coronary angiography: a prospective, multicenter, multivendor study. *Journal of the American College of Cardiology* 2008; **52**(25): 2135-44.
38. Meijboom WB, Mollet NR, Van Mieghem CA, et al. Pre-operative computed tomography coronary angiography to detect significant coronary artery disease in patients referred for cardiac valve surgery. *Journal of the American College of Cardiology* 2006; **48**(8): 1658-65.
39. Meijboom WB, Mollet NR, Van Mieghem CA, et al. 64-Slice CT coronary angiography in patients with non-ST elevation acute coronary syndrome. *Heart* 2007; **93**(11): 1386-92.
40. Meijboom WB, Weustink AC, Pugliese F, et al. Comparison of diagnostic accuracy of 64-slice computed tomography coronary angiography in women versus men with angina

pectoris. *American Journal of Cardiology* 2007; **100**(10): 1532-7.
41. Mendoza V, Llerena L, Llerena L, Rodríguez L, Olivares E, Linares R. Ischemic heart disease diagnosed by 64 slice computed tomography coronary angiography. *The Internet Journal of Cardiology* 2009; **7**(1).
42. Mollet NR, Cademartiri F, van Mieghem CA, et al. High-resolution spiral computed tomography coronary angiography in patients referred for diagnostic conventional coronary angiography. *Circulation* 2005; **112**(15): 2318-23.
43. Nikolaou K, Knez A, Rist C, et al. Accuracy of 64-MDCT in the diagnosis of ischemic heart disease. *AJR American journal of roentgenology* 2006; **187**(1): 111-7.
44. Nikolaou K, Rist C, Wintersperger BJ, et al. Clinical value of MDCT in the diagnosis of coronary artery disease in patients with a low pretest likelihood of significant disease. *AJR American journal of roentgenology* 2006; **186**(6): 1659-68.
45. Ovrehus KA, Jensen JK, Mickley HF, et al. Comparison of usefulness of exercise testing versus coronary computed tomographic angiography for evaluation of patients suspected of having coronary artery disease. *The American journal of cardiology* 2010; **105**(6): 773-9.
46. Ovrehus KA, Munkholm H, Bottcher M, Botker HE, Norgaard BL. Coronary computed tomographic angiography in patients suspected of coronary artery disease: impact of observer experience on diagnostic performance and interobserver reproducibility. *Journal of cardiovascular computed tomography* 2010; **4**(3): 186-94.
47. Pontone G, Andreini D, Ballerini G, Nobili E, Pepi M. Diagnostic work-up of unselected patients with suspected coronary artery disease: complementary role of multidetector computed tomography, symptoms and electrocardiogram stress test. *Coronary artery disease* 2007; **18**(4): 265-74.
48. Pontone G, Andreini D, Bartorelli AL, et al. Feasibility and accuracy of a comprehensive multidetector computed tomography acquisition for patients referred for balloon-expandable transcatheter aortic valve implantation. *American heart journal* 2011; **161**(6): 1106-13.
49. Pontone G, Andreini D, Bartorelli AL, et al. Diagnostic accuracy of coronary computed tomography angiography: a comparison between prospective and retrospective electrocardiogram triggering. *Journal of the American College of Cardiology* 2009; **54**(4): 346-55.
50. Pontone G, Andreini D, Quaglia C, Ballerini G, Nobili E, Pepi M. Accuracy of multidetector spiral computed tomography in detecting significant coronary stenosis in patient populations with differing pre-test probabilities of disease. *Clinical radiology* 2007; **62**(10): 978-85.
51. Pouleur AC, le Polain de Waroux JB, Kefer J, et al. Usefulness of 40-slice multidetector row computed tomography to detect coronary disease in patients prior to cardiac valve surgery. *European radiology* 2007; **17**(12): 3199-207.
52. Pouleur AC, le Polain de Waroux JB, Kefer J, Pasquet A, Vanoverschelde JL, Gerber BL. Direct comparison of whole-heart navigator-gated magnetic resonance coronary angiography and 40- and 64-slice multidetector row computed tomography to detect the coronary artery stenosis in patients scheduled for conventional coronary angiography. *Circulation Cardiovascular imaging* 2008; **1**(2): 114-21.
53. Pugliese F, Mollet NR, Runza G, et al. Diagnostic accuracy of non-invasive 64-slice CT coronary angiography in patients with stable angina pectoris. *European radiology* 2006; **16**(3): 575-82.
54. Rixe J, Rolf A, Conradi G, et al. Detection of relevant coronary artery disease using dual-source computed tomography in a high probability patient series: comparison with invasive angiography. *Circulation journal : official journal of the Japanese Circulation Society* 2009; **73**(2): 316-22.
55. Rochitte CE, George RT, Chen MY, et al. Computed tomography angiography and perfusion to assess coronary artery stenosis causing perfusion defects by single photon emission computed tomography: the CORE320 study. *European heart journal* 2014; **35**(17): 1120-30.
56. Sato A, Nozato T, Hikita H, et al. Incremental value of combining 64-slice computed tomography angiography with stress nuclear myocardial perfusion imaging to improve noninvasive detection of coronary artery disease. *Journal of nuclear cardiology : official publication of the*

American Society of Nuclear Cardiology 2010; **17**(1): 19-26.
57. Scheffel H, Alkadhi H, Plass A, et al. Accuracy of dual-source CT coronary angiography: First experience in a high pre-test probability population without heart rate control. *European radiology* 2006; **16**(12): 2739-47.
58. Shabestari AA, Abdi S, Akhlaghpoor S, et al. Diagnostic performance of 64-channel multislice computed tomography in assessment of significant coronary artery disease in symptomatic subjects. *The American journal of cardiology* 2007; **99**(12): 1656-61.
59. Sun K, Han RJ, Cui LF, et al. Feasibility and Diagnostic Accuracy for Assessment of Coronary Artery Stenosis of Prospectively Electrocardiogram-gated High-pitch Spiral Acquisition Mode Dual-source CT Coronary Angiography in Patients with Relatively Higher Heart Rates: in Comparison with Catheter Coronary Angiography. *Chinese medical sciences journal = Chung-kuo i hsueh k'o hsueh tsa chih* 2013; **27**(4): 213-9.
60. Ugolini P, Pressacco J, Lesperance J, et al. Evaluation of coronary atheroma by 64-slice multidetector computed tomography: Comparison with intravascular ultrasound and angiography. *The Canadian journal of cardiology* 2009; **25**(11): 641-7.
61. Watkins MW, Hesse B, Green CE, et al. Detection of coronary artery stenosis using 40-channel computed tomography with multi-segment reconstruction. *The American journal of cardiology* 2007; **99**(2): 175-81.
62. Xu L, Yang L, Fan Z, Yu W, Lv B, Zhang Z. Diagnostic performance of 320-detector CT coronary angiography in patients with atrial fibrillation: preliminary results. *European radiology* 2011; **21**(5): 936-43.
63. Yang L, Zhang Z, Fan Z, et al. 64-MDCT coronary angiography of patients with atrial fibrillation: influence of heart rate on image quality and efficacy in evaluation of coronary artery disease. *AJR American journal of roentgenology* 2009; **193**(3): 795-801.
64. Unpublished study #1.
65. Unpublished study #2.

Supplementary table 18

**Studies without core labs**		**Studies with core labs**	
**Sensitivity (95% CI)**	**Specificity (95% CI)**	**Sensitivity (95% CI)**	**Specificity (95% CI)**
95.2 (92.5 to 96.9)	80.8 (76.7 to 84.3)	73.9 (39.4 to 92.5)	61.7 (40.9 to 78.9)

Supplementary figure 1 | Proportion of studies with low, high or unclear risk of bias in QUADAS-2

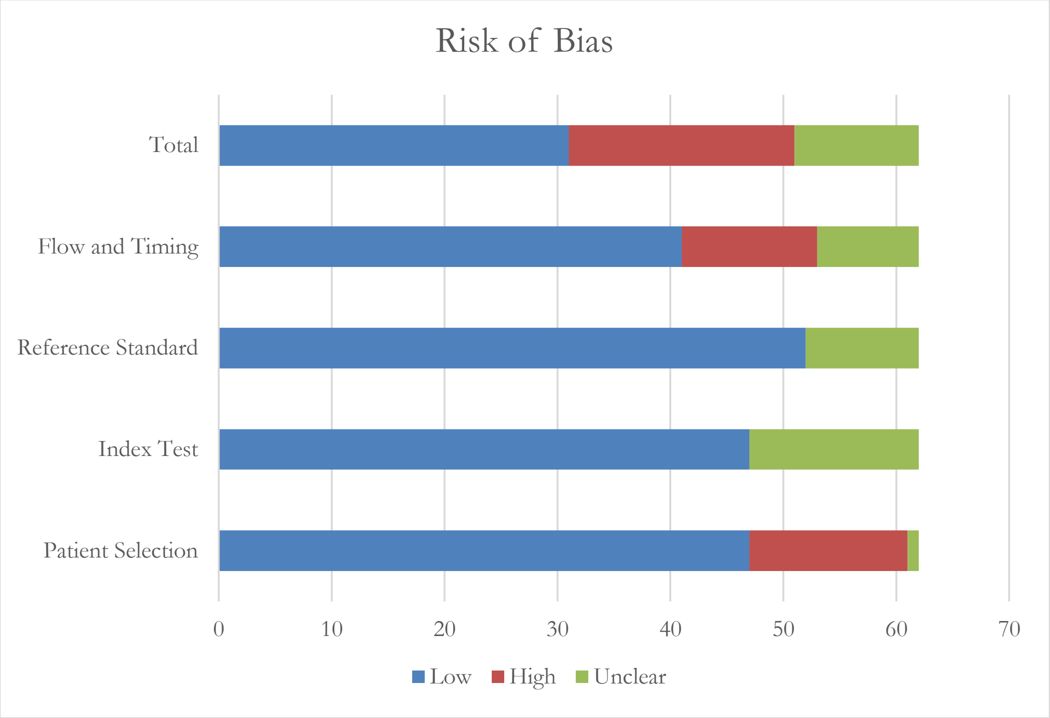

The X-axis displays the proportional rate of studies (in %) bearing a low (blue), high (red) or unclear (green) risk of bias regarding the items displayed on the Y-axis. In general, the risk of bias was low, especially regarding the both diagnostic tests, while it was highest in patient selection.

Supplementary figure 2 | Proportion of studies with low, high or unclear concerns regarding applicability in QUADAS-2

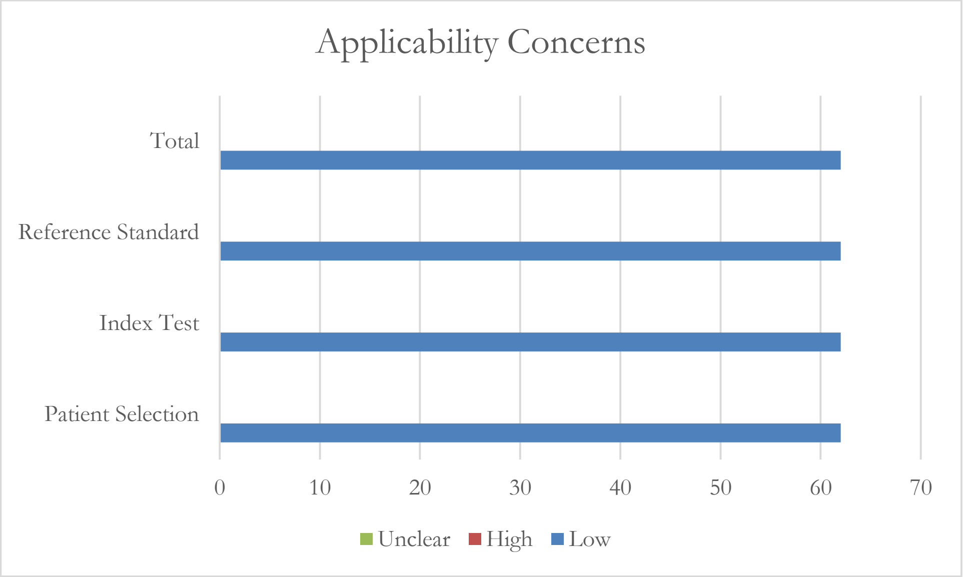

The X-axis displays the proportional rate of studies (in %) bearing a low (blue), high (red) or unclear (green) risk of bias regarding the items displayed on the Y-axis. There are only low concerns regarding the applicability of studies included in the analysis.

Supplementary figure 3| Clinical diagnostic performance of CTA as a function of pretest probability excluding non-diagnostic examinations

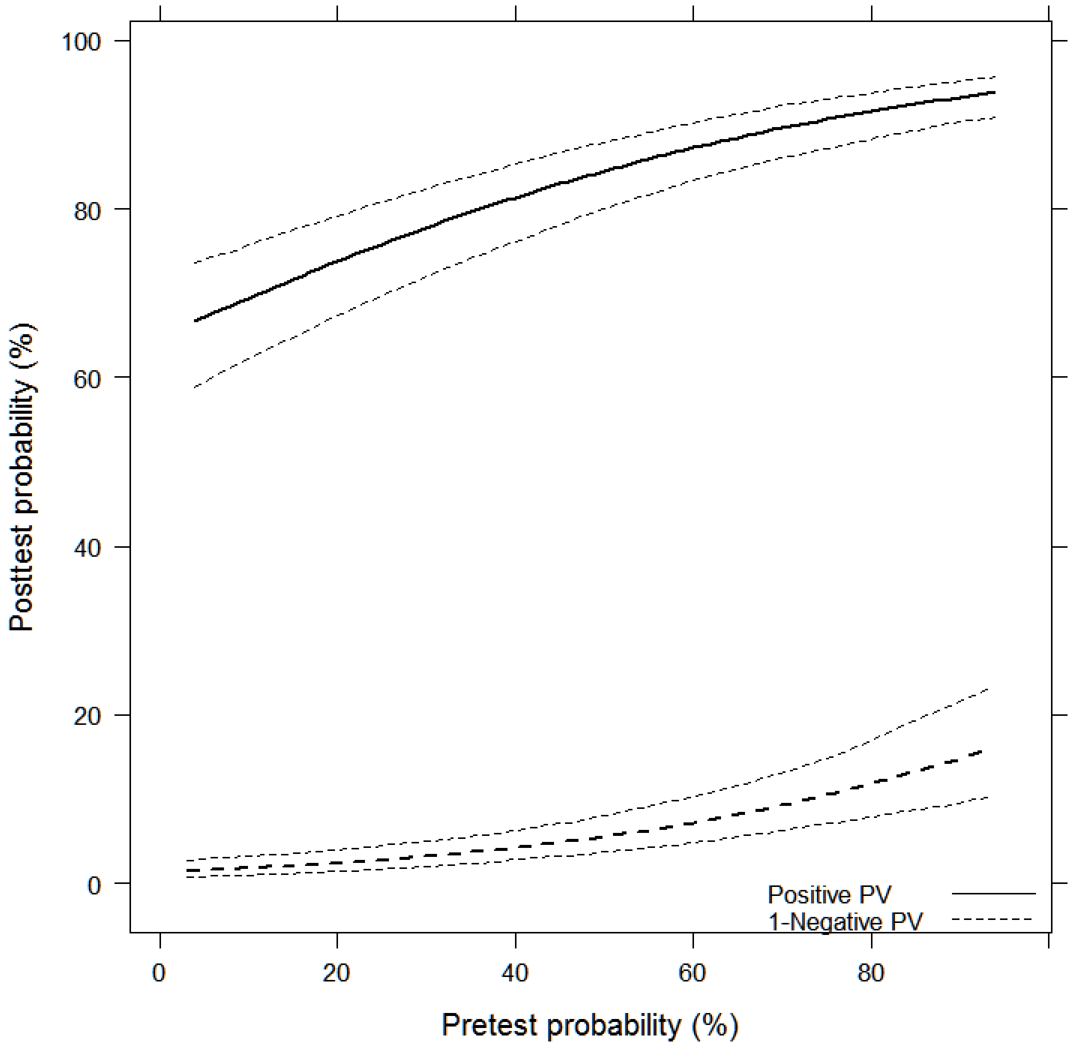

The X-axis represents the predicted clinical pretest probability and the Y-axis shows the positive predictive value (PV) and 1-Negative PV with their 95% CI based on the generalised linear mixed model excluding non-diagnostic CTA examinations. Disease probabilities were predicted by averaging over the random-effects distribution.

Supplementary figure 4 | Heart rate during CTA in female and male patients

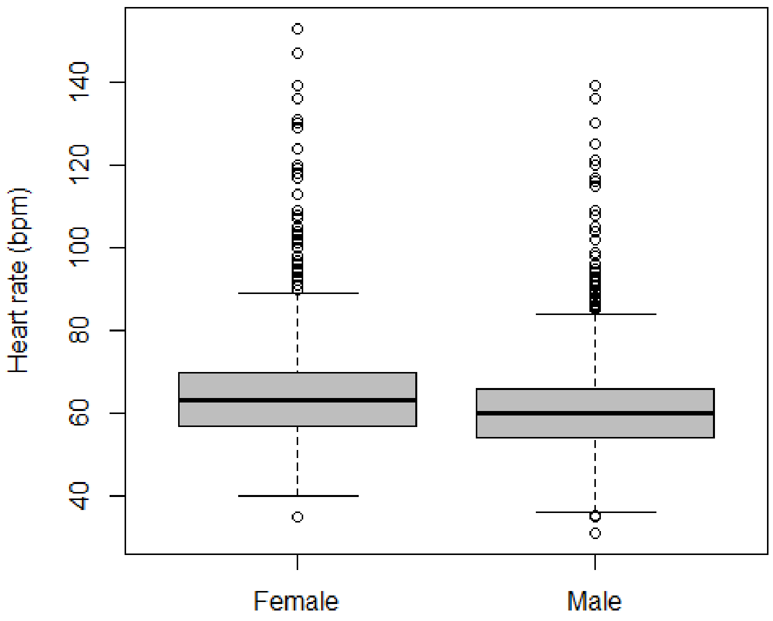

Median heart rate was significantly higher in females during CTA (63 beats per minute [bpm], IQR: 57-70) compared with males (60 bpm, IQR: 54-66, MWM test: W = 3504600, p<0.001). Data on heart rate were missing for 62 of 1859 females and 151 of 3473 males.

Supplementary figure 5 | Coronary calcium score in patients with up to 75 years of age vs older patients

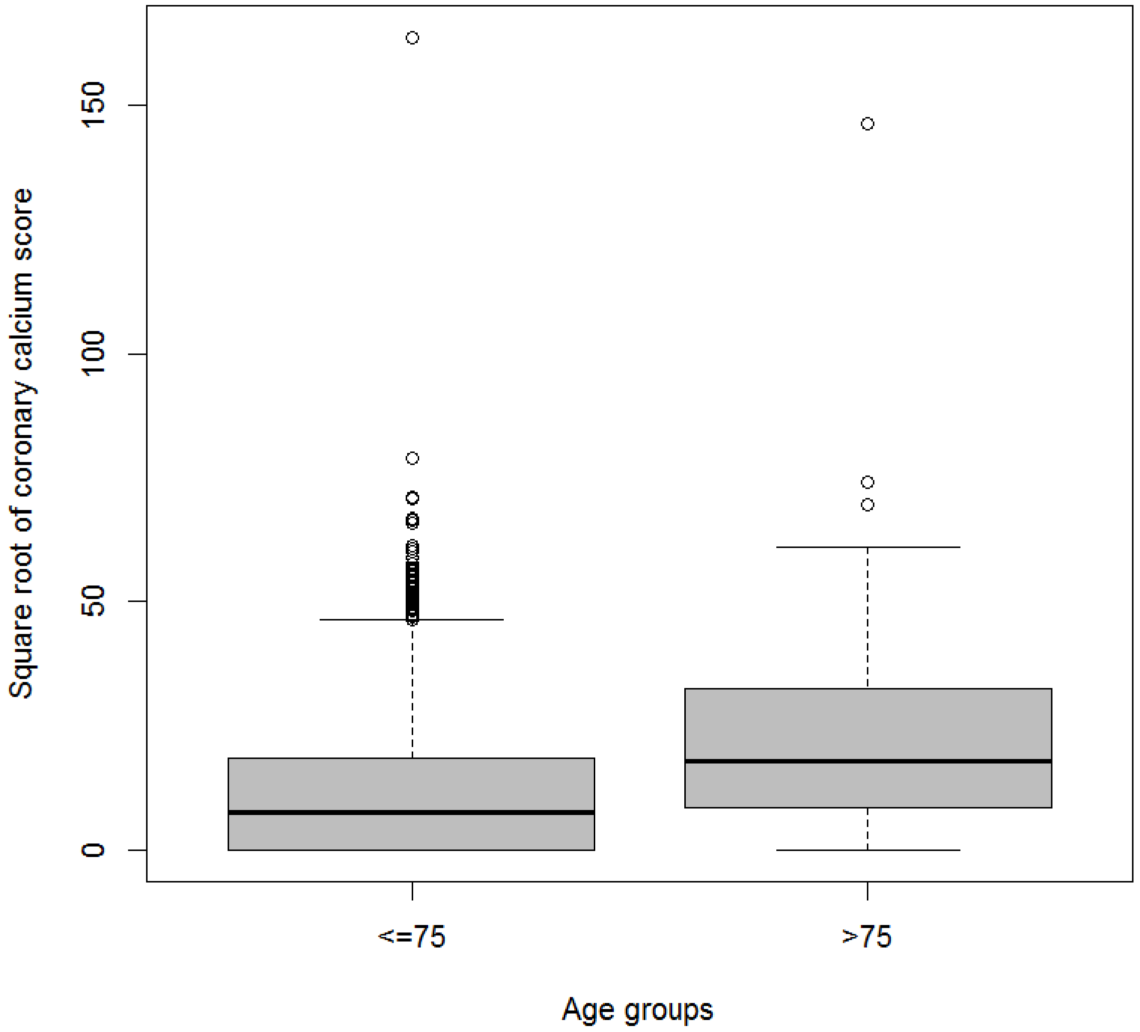

Median calcium score (square root transformed) was significantly higher in patients above 75 years of age (17.916, IQR: 8.367-32.348) compared with younger patients (7.483, IQR: 0.000-13.74, p<0.001). Data on calcium score were missing for 311 of 552 patients above 75 years of age and 2567 of 4780 younger patients. The boxplot shows square root-transformed data of coronary calcium scores because of skewness of data.

	**Square root transformed data**						
**Groups**	Min	1st Qu.	Median	Mean	3rd Qu.	Max	NA's
Age ≤75y	0.000	0.000	7.483	11.928	13.574	163.677	2567
Age >75y	0.000	8.367	17.916	21.916	32.348	146.595	311

Supplementary figure 6 | Receiver operating characteristic curves for CTA by subgroup excluding non-diagnostic CT examinationas

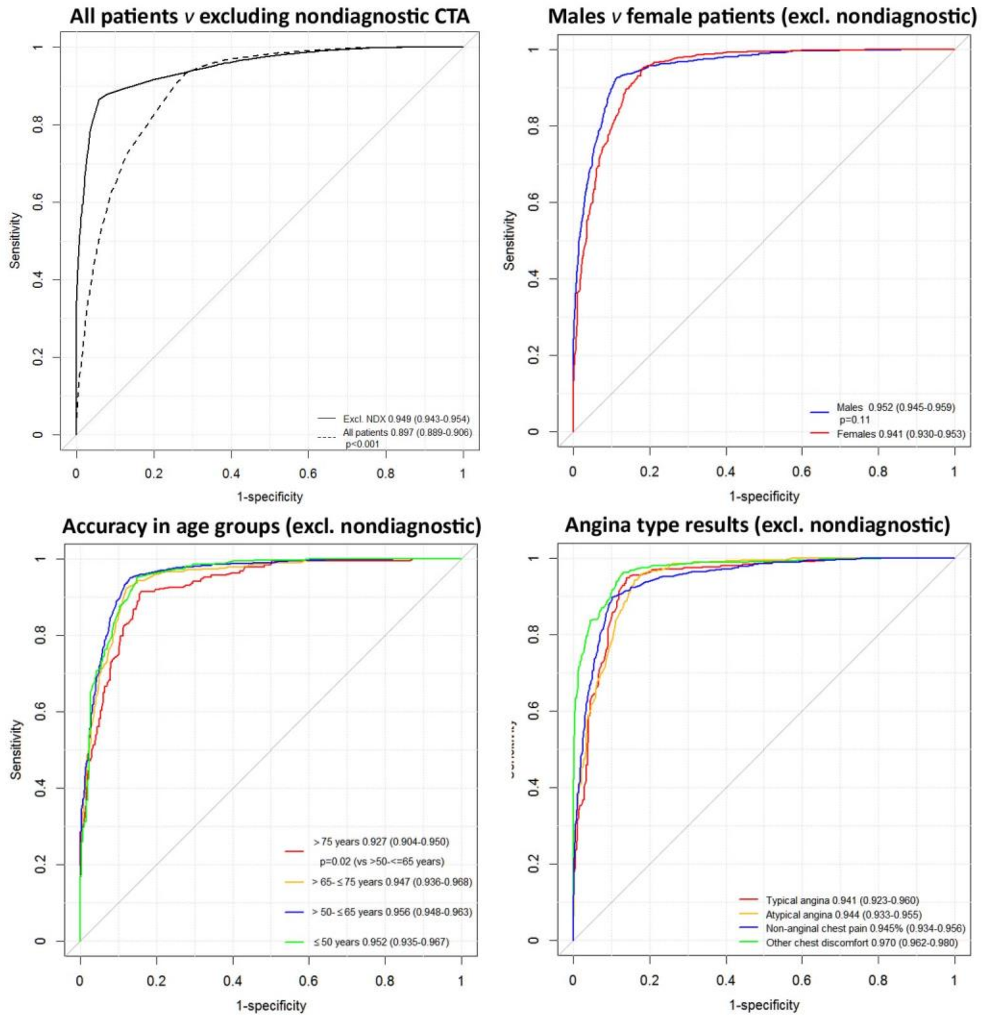

Subgroup comparisons in the three panels are provided for all patients after exclusion of non-diagnostic CT examinations. In the upper left panel diagnostic performance results are shown for all patients in comparison to results obtained after exclusion of non-diagnostic (NDX) test results like also demonstrated in the manuscript. Considering all patients resulted in lower performance, which is a more accurate prediction of the real-world performance to be expected. In contrast to the manuscript subgroup comparisons in the other three panels here are provided for patients after exclusion of non-diagnostic CT examinations: diagnostic performance is now similar in females and males. The other comparisons revealed similar results as when including non-diagnostic CT examinations: CTA's accuracy was lower in patients older than 75, and angina pectoris types were not significantly associated with performance. Like in the manuscript curves were generated using a generalised linear mixed model and predictions based on these models. Computations were performed with the statistical package R and the packages lme4 and pROC. AUC were constructed using the observed data and model-based predictions, which also included the random effects reflecting variability between studies and unobserved influential variables.

Supplementary figure ~~6~~ 7 | Publication bias analysis using Funnel plot

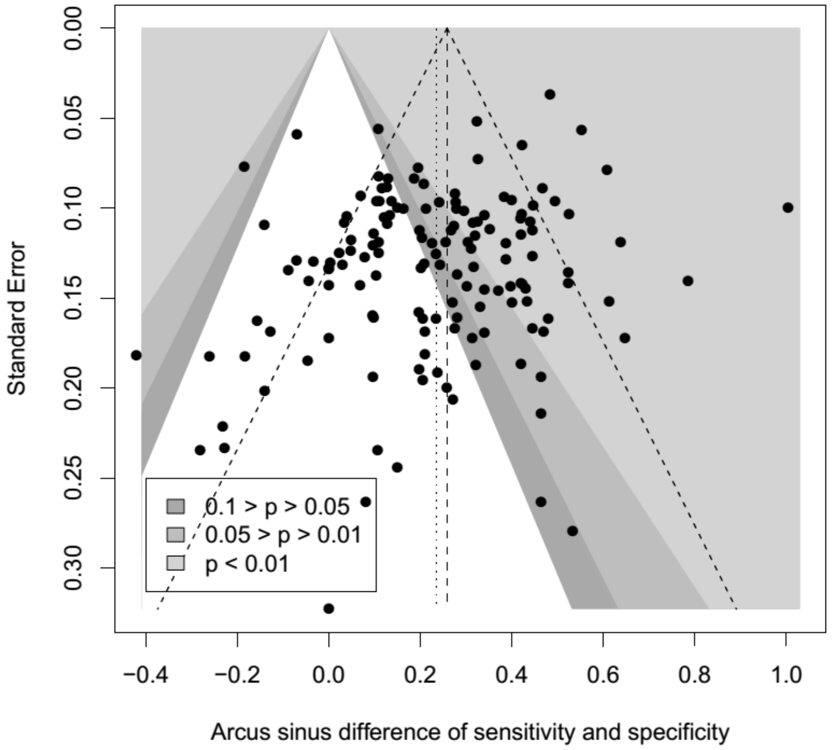

This funnel plot shows the arc sinus difference of sensitivity and specificity *vs.* the corresponding standard error. The corresponding statistical test was performed using the method proposed by Rücker with a rank test: Kendal's tau = -661 s.e. = 584.973, z= -1.13, p=0.2585. There was no sign of publication bias.

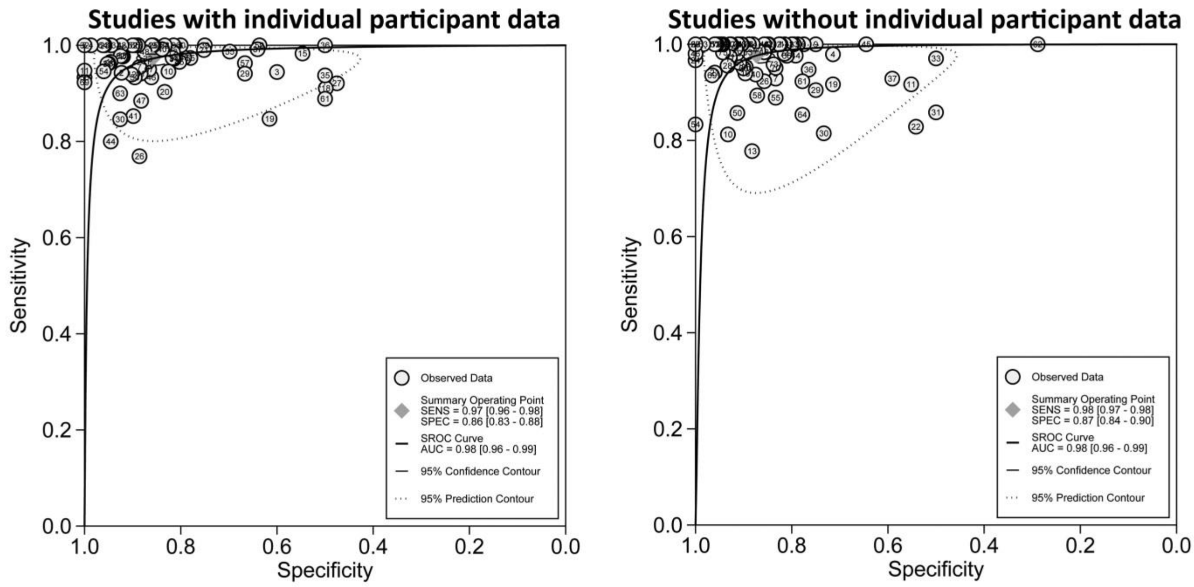

Supplementary figure 8~~7~~ | Summary receiver operating characteristic curves for CTA studies with and without individual participant data available

Curves are shown for studies with individual participant data (IPD) available in comparison to studies for which no IPD were available. Curves were calculated using aggregated data methodology (summary receiver operating characteristic curves) both for panels and after excluding non-diagnostic test results, which were not consistently available in publications of studies which did not provide individual participant data. Among the 76 studies which provided individual participant data, aggregate data were not available for seven studies, two of them unpublished, leaving 69 for the analysis of studies with IPD, while 76 of the 78 studies which did not provide IPD had aggregate data available (see figure 1 of main paper). There was no significant difference in diagnostic performance between these two groups of diagnostic accuracy studies (P=0.73). Further details are shown in table 4 of main paper.

Supplementary PRISMA item 1 | PICOS

Patients:	Patients with stable chest discomfort and a clinical indication to undergo invasive coronary angiography
Intervention:	Coronary computed tomography angiography
Comparison/Control:	Invasive coronary angiography
Outcomes:	Diagnostic accuracy as defined by positive and negative predictive values as a function of pretest probability and sensitivity and specificity
Setting:	Individual participant meta-analysis of diagnostic accuracy studies comparing coronary computed tomography angiography with invasive coronary as the reference standard.

Supplementary PRISMA items 2 | Search strategy for searching PubMed via Medline
(("tomography, x-ray computed"[MeSH Terms])OR ("computed tomography"[Text Words])OR ("CT"[Text Words])OR ("multidetector"[Text Words])OR ("multi-detector"[Text Words])OR ("multi detector"[Text Words])OR ("MDCT"[Text Words])OR ("multislice"[Text Words])OR ("multi-slice"[Text Words])OR ("MSCT"[Text Words])OR ("dual-source"[Text Words])OR ("dual source"[Text Words]) OR ("DSCT"[Text Words])OR ("multi-row"[Text Words])OR ("multi row"[Text Words]))AND(("coronary angiography"[Mesh Terms])OR ("coronary angiography"[Text Words]))AND(("coronary disease"[Mesh Terms])OR ("coronary artery disease"[Mesh Terms])OR ("coronary stenosis"[Mesh Terms])OR ("coronary disease"[Text Words])OR ("coronary artery disease"[Text Words])OR ("coronary stenosis"[Text Words])OR ("CAD"[Text Words])OR ("coronary heart disease"[Text Words])OR ("CHD"[Text Words]))
Further details have been reported in the study protocol.

Supplementary PRISMA items 3 | Data items and IPD collection file
Data items were predefined and collected using an IPD collection in a Microsoft Excel format that was sent to all corresponding authors of identified eligible diagnostic accuracy studies. Data items consisted of all data necessary to estimate patients' pretest probability (age, gender, angina pectoris classification), 3x2 cross tabulations (test positive, negative or non-diagnostic for both, coronary computed tomography angiography and invasive coronary angiography), details of each CT scan (number of detector rows, heart rate during scan) and patient information on cardiac medical history (stents or bypasses received, risk factors, stress tests performed). Please find the IPD collection file for all collected data items in Appendix C.

Supplementary PRISMA items 4 | Data revision process
Data harmonisation was performed by two independent readers who analysed data and searched for non-plausible data, including range checks, wrong entries, non-logical values and date checks etc. The two readers also recalculated 2x2 and/or 3x2 cross tabulations and compared these with the published data if possible and if available, non-diagnostic examinations were also recorded. After consensus the data management team solved contradictions. If not possible, new Excel files were created for the missing data and again sent to the corresponding author with comments on implausible data and the request to check. For any remaining implausibilities, further reminder mails were sent. Most revisions had to be done on angina type classification (dual or missing entries, rare cases of other classification), missing values for heart rate during CT, 3x2 cross tabulations (due to another definition of non-diagnostic examinations), missing values of CT characteristics, and typing errors.
