# Supplementary material for: Diagnosis of obstructive coronary artery disease using computed tomography angiography in patients with stable chest pain depending on clinical probability and in clinically important subgroups: meta-analysis of individual patient data
Source: BMJ. 2019 Jun 12;365:l1945. doi: 10.1136/bmj.l1945 (PMC6561308; doi:10.1136/bmj.l1945)
Supplement: Supplementary file 3 — Web appendix 3: IPD collection file [file haar046861.ww3.pdf]

Please use 0=no; 1=yes if not otherwise indicated

Patient No.	Date of Birth	Date of Inclusion	Gender	Typical Angina Pectoris	Atypical Angina Pectoris	Non-anginal Chest Pain	Other Chest Discomfort	Pain Unknown	Heart Rate During CT (bpm)	**CT**	Positive	Negative	Nondiagnostic	Comment on Nondiagnostic
Comments and Explanations			Female=0 Male=1	Typical Angina Pectoris: 1. typical type of pain and 2. getting worse when exercising and 3. getting better during rest and/or nitro.	Atypical Angina Pectoris=2 out of the 3 criteria for Typical Angina	Non-anginal Chest Pain=1 out of the 3 Criteria for Typical Angina	Other Chest Discomfort=0 out of the 3 Criteria for Typical Angina		Average heart rate during scanning		At least one coronary artery with at least one 50% diameter stenosis.	No coronary artery with at least one 50% diameter stenosis.	Nondiagnostic: if no stenosis is seen and at least one vessel is not fully evaluable	Please give reason why a patient was nondiagnostic, e.g., motion artifact, too much calcium etc.
Patient Example (67 years, male, true positive in CT with atypical angina. Please start your patients on the following line.)	07.03.1945	14.03.2001	1	0	1	0	0	0	55		1	0	0	

Patient No.	**CATH**	Positive	Negative	Nondiagnostic	Comment on Nondiagnostic	**Additional CT Characteristics**	DLP (mGy*cm)	mSv	Retrospective (=0) or Prospective (=1) Scanning	Number of Detector Rows Used	Oral Beta Blockade as Part of the CT Protocol	Type of Oral Beta Blocker	Dose of Oral Beta Blocker (mg)	IV Beta Blockade	Type of IV Beta Blocker	Dose of IV Beta Blocker (mg)	Sublingual Nitroglyerin	Dose of Sublingual Nitroglyerin (mg)
Comments and Explanations		At least one coronary artery with at least one 50% diameter stenosis.	No coronary artery with at least one 50% diameter stenosis.	Nondiagnostic: if no stenosis is seen and at least one vessel is not fully evaluable	Please give reason why a patient was nondiagnostic, e.g., no selective coronary injection possible	The following information is nice to have, and everyone contributing will be an author on the resulting ancillary study but this is not a must for the primary analysis. Please provide the available information.	Please provide DLP or mSv											
Patient Example (67 years, male, true positive in CT with atypical angina. Please start your patients on the following line.)		1	0	0				5	0	12	1	Atenolol	150	1	Esmolol	200	1	1,2

					The following information is nice to have, and everyone contributing will be an author on the resulting ancillary study but this is not a must for the primary analysis. Please provide the available information.	0=CATH afterwards; 1=CATH before CT		The following information is nice to have, and everyone contributing will be an author on the resulting ancillary study but this is not a must for the primary analysis. Please provide the available information.				yes=1 if rest angina (>20 min) within the last week, severe new-onset angina (at least CCS III that started in the last 2 months), or increasing angina (increased by at least 1 CCS class to CCS III in the last 2 months).										The following information is nice to have, and everyone contributing will be an author on the resulting ancillary study but this is not a must for the primary analysis. Please provide the available information.									
Contrast Agent Amount (ml)	Type of Contrast Agent	Contrast Agent Flow (ml/s)	Contrast Agent Concentration (Iodine mg/ml)	Breathhold Duration (s)	**Additional CATH Characteristics**	CATH after or before CT?	Days between CT and CATH	**Additional Patient Characteristics**	Weight (kg)	Height (cm)	BMI	Unstable Angina	Coronary Stents Present	Coronary Bypasses Present	Hypertension	Diabetes	Hyperlipidemia	Current Smoker	Former Smoker	Positive Family History	Prior Myocardial Infarction	**Additional Tests Characteristics**	Rest ECG Done	Rest ECG: ST Changes	Rest ECG: Q waves	Stress ECG Done	Stress ECG: Significant ST Changes	Stress Echo Done	Stress Echo: Significant Signs of Ischemia	Stress SPECT Done	Stress SPECT: Significant Signs of Ischemia
80	Iodixanol	5	320	8		0	1		85	174	28	0	0	0	1	0	1	0	1	1	1		1	1	1	1	1	0	0	0	0
